# α-Photooxygenation of chiral aldehydes with singlet oxygen

**DOI:** 10.3762/bjoc.15.205

**Published:** 2019-08-30

**Authors:** Dominika J Walaszek, Magdalena Jawiczuk, Jakub Durka, Olga Drapała, Dorota Gryko

**Affiliations:** 1 Institute of Organic Chemistry, Polish Academy of Sciences, Kasprzaka 44/52, 01-224 Warsaw, Poland; 2 Centre of New Technologies, University of Warsaw, Banacha 2c, 02-097 Warsaw, Poland; 3 Department of Chemistry, Warsaw University of Technology, Noakowskiego 3, 00-664 Warsaw, Poland

**Keywords:** 1,2-diols, ECD, enamines, organocatalysis, porphyrins, silyl ethers of diarylprolinols, singlet oxygen

## Abstract

Organocatalytic α-oxygenation of chiral aldehydes with photochemically generated singlet oxygen allows synthesizing chiral 3-substituted 1,2-diols. Stereochemical results indicate that the reaction in the presence of diarylprolinol silyl ethers is highly diastereoselective and that the configuration of a newly created stereocenter at the α-position depends predominantly on the catalyst structure. The absolute configuration of chiral 1,2-diols has been unambiguously established based on electronic circular dichroism (ECD) and TD-DFT methods.

## Introduction

Carbonyl compounds are one of the most important building blocks in organic synthesis. As a consequence, there is a constant need for new methodologies enabling their functionalization, particularly in a stereoselective manner. Among them, asymmetric α-oxygenation of aldehydes still represents a challenging task. Most efficient methods require simultaneous use of chiral amines or Brønsted acids, and harsh oxidants like nitrosobenzene [1–3], TEMPO [4], or benzoyl peroxide [5–8]. Therefore, the use of environmentally friendly reagents instead is highly desirable. Along this line, singlet oxygen by being easily photochemically generated from triplet oxygen in the presence of organic dyes seems promising. Despite its high reactivity and small molecule size, there are few examples of its use not only in diastereoselective synthesis but also in enantioselective reactions [9–10].

**Scheme 1:**
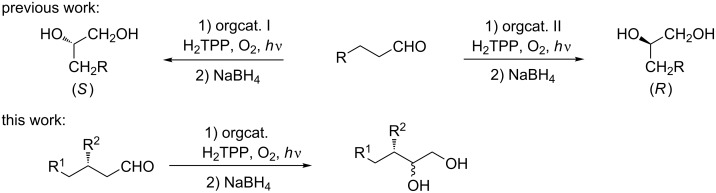
Asymmetric α-photooxygenation of chiral aldehydes.

Inspired by Cόrdova’s work [11–13], we explored the idea of merging enamine catalysis with photocatalytic oxygenation with singlet oxygen for α-hydroxylation of aldehydes [14–15]. Recently, we have reported that organocatalytic photooxygenation of aldehydes affords the desired diols (after in situ reduction) in decent yield with either (*R*)- or (*S*)-selectivity depending on a catalysts used [14]. In the presence of prolinamides the (*R*)-enantiomer predominates while the imidazolidinone-catalyzed reaction is (*S*)-enantioselective. Nevertheless, the scope is limited to simple, achiral aldehydes. As the synthesis of more complex targets often requires functionalization of molecules with stereocenters being already installed, we wondered whether and how preexisting stereochemical elements influence the stereoselectivity of this reaction. To this end we investigated α-photooxygenation of chiral aldehydes with a stereocenter at the β-position (Scheme 1). In such a case, the outcome should depend on the relationships between the absolute configuration of the starting material and the catalyst.

## Results and Discussion

Our previous studies on α-photooxygenation of achiral aldehydes with ^1^O_2_ in the presence of chiral amines supported by DFT calculations indicate that the reaction is highly enantioselective only when both enamine structural fragments (substituents originating from an aldehyde and an organocatalyst) interact with each other in such a way that one side of the enamine is predominantly shielded from singlet oxygen. As a result, small alterations to the aldehyde structure require detailed optimization of the catalyst structure [14]. Preliminary data for α-photooxygenation of chiral aldehydes suggested that the stereocenter at the β-position has a strong impact on the stereoselectivity level.

### Model reaction

**Scheme 2:**
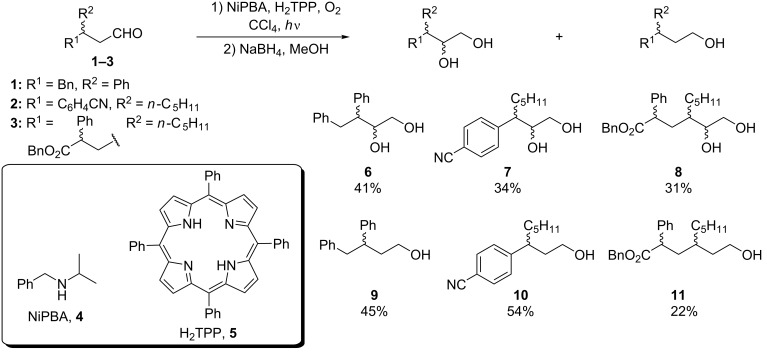
α-Photooxygenation of β-substituted aldehydes.

Firstly, we tested three aldehydes **1**–**3** bearing substituents at the β-position. These substrates are easily accessible by photochemical means, according to the procedures reported by the groups of Melchiorre or MacMillan [16–18]. Preliminary experiments showed that photooxygenation of chiral aldehydes **1**, **2** or **3** (used **1** as *S*/*R* 2:1 mixture of enantiomers, **2** as racemate, and **3** as 1.4:1 mixture of diastereoisomers) in the presence of *N*-isopropylbenzylamine (NiPBA, **4**) as an organocatalyst and *meso*-tetraphenylporphyrin (H_2_TPP, **5**) as a photosensitizer followed by in situ reduction with NaBH_4_, proceeded similarly to the reported results for simple, achiral aldehydes giving the desired diols **6**–**8** in 31–41% yields with moderate conversion and alcohols **9**–**11** as byproducts (Scheme 2) [14].

Photooxygenation of 3,4-diphenylbutanal (**1**) affording the desired diol **6** accompanied by alcohol **9** as the only byproduct was chosen as a model reaction for optimization studies. From a practical point of view it was important that diastereoisomers *syn*-**6** and *anti*-**6** could be separated by column chromatography.

### Photooxygenation of 3,4-diphenylbutanal (**1**) with chiral organocatalysts

**Scheme 3:**
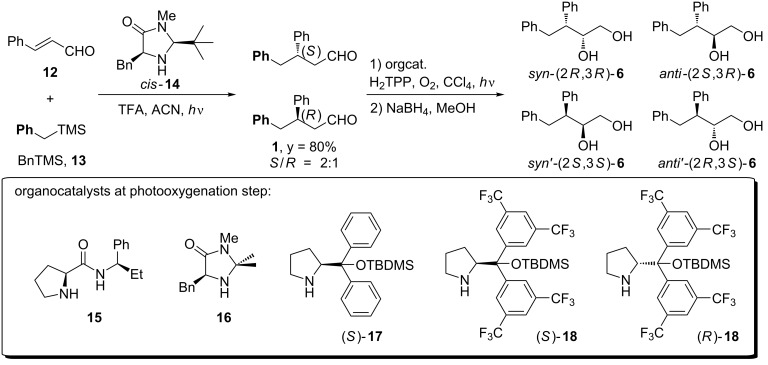
Synthesis and α-photooxygenation of 3,4-diphenylbutanal (**1**).

The highly enantioselective synthesis of 3,4-diphenylbutanal (**1**), according to the procedure developed by Melchiorre, requires the use of noncommercially available sterically bulky silyl ethers [16]. For that reason, for our optimization studies we used enantioenriched aldehyde **1** (*S*/*R* 2:1 mixture of enantiomers) formed in the photochemical reaction of cinnamaldehyde (**12**) with benzyltrimethylsilane (BnTMS, **13**) catalyzed by commercially available imidazolidinone *cis*-**14** (Scheme 3) [16]. Aldehyde **1** was subjected to photooxygenation in the presence of various organocatalysts: amide **15**, imidazolidinone **16**, diarylprolinol silyl ethers (*S*)-**17** and (*S*)-**18** which proved the most effective in the photooxygenation of achiral 3-phenylpropanal (Table 1) [14]. The prolinamide-catalyzed reaction furnished only small amounts of the desired product **6** (Table 1, entry 1), higher yields were obtained in the presence of imidazolidinone **16** and silyl ether (*S*)-**17**, 31% and 52%, respectively (Table 1, entries 2 and 3). The yield further increased to 59% upon the addition of phosphate buffer. The highest level of stereoselectivity was enforced by bulkier and electron-withdrawing groups as diarylprolinol silyl ether **18** (Table 1, entries 4 and 5).

**Table 1:** Stereoselectivity of α-photooxygenation reaction of 3,4-diphenylbutanal (**1**).

Entry	Cat.	Yield [%]	dr (*syn:anti*)	er (*syn:syn'*)	er (*anti:anti'*)	major (conf.)	minor (conf.)

1	**15**	<10	n/a	n/a	n/a	*–*	*–*
2	**16**	31	1:2	20:80	90:10	*anti* (2*S*,3*R*)	*syn’* (2*S*,3*S*)
3	(*S*)-**17**	52 (59)^a^	1:2	19:81	91:9	*anti* (2*S*,3*R*)	*syn’* (2*S*,3*S*)
4	(*S*)-**18**	35	1:2	13:87	87:13	*anti* (2*S*,3*R*)	*syn’* (2*S*,3*S*)
5	(*R*)-**18**	40	2:1	96:4	4:96	*syn* (2*R*,3*R*)	*anti’* (2*R*,3*S*)

^a^Phosphate buffer pH 7 was used as an additive.

**Scheme 4:**
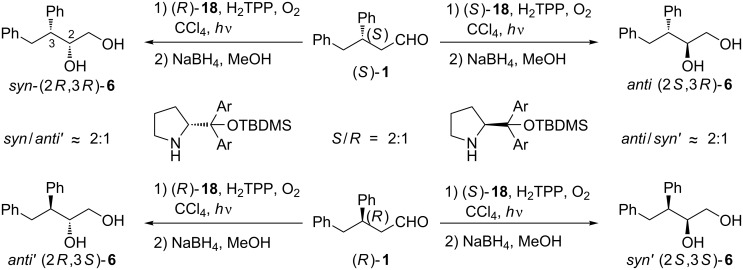
Stereoselective α-photooxygenation of 3,4-diphenylbutanal (**1**) with ^1^O_2_.

The absolute configuration of diastereoisomers formed was assigned using chiroptical spectroscopic methods (see section ‘Determination of the absolute configuration of the product by electronic circular dichroism spectroscopy’). Functionalization of 3,4-diphenylbutanal (**1**) at the α-position can lead to four stereoisomers, but photooxygenation catalyzed by secondary amines **16**, **17** and **18** furnished only two of them. The diastereoisomeric ratio was always close to 2:1, the same as er for enantioenriched substrate **1** thus indicating that the organocatalytic photooxygenation of aldehydes with singlet oxygen is highly diastereoselective. In the case, when two chiral compounds participate in a given reaction, they, according to Masamune, may be considered as matching or mismatching pairs [19]. As a consequence, an increased or decreased level of stereoselectivity can be observed. In our case, enantiomeric catalysts (*S*)-**18** or (*R*)-**18** impose the same level of stereoselectivity but with the opposite direction (Scheme 4).

No significant match/mismatch effect was observed as catalyst (*S*)-**18** provided two diasteroisomers with (*S*)-configuration of the newly created stereocenter in high predominance, while the reaction catalyzed by prolinol derivative (*R*)-**18** almost exclusively led to (2*R*)-diols. These results indicate that photooxygenation of chiral aldehydes with singlet oxygen provides the desired product and the configuration of the newly created stereocenter depends mainly on the catalyst used. There is no doubt that the presence of the phenyl substituent at the β-position of the aldehyde is necessary to ensure a high level of selectivity (by effective shielding), but on the other hand the ability to alter the configuration of a newly created stereocenter by changing only the catalyst configuration is a quite unprecedented phenomenon in singlet oxygen reactions. Usually in those reactions the introduction of a steric hindrance much larger than the phenyl ring (i.e., adamantyl) or a chiral auxiliary (i.e., oxazolidinone) into the substrate structure is required for highly stereoselective reactions [20–22].

### ‘One-pot’ photochemical α,β-functionalization of cinnamaldehyde

Over the last few years, photochemical methods for asymmetric functionalisation of carbonyl compounds at either α or β-position has been of particular interest [23]. Just to mention Córdova’s α-oxygenation [11–13] or β-alkylation or β-arylation reported by Melchiorre [16] and MacMillan [17–18]) which represents only a tip of an iceberg of photochemical methods for the introduction of substituents into an aldehydes’ structure. Interestingly, to the best of our knowledge, there are no reports on ‘one-pot’ reactions leading to difunctionalization at both α and β-positions. To this end, in subsequent experiments, we attempted to merge two photochemical processes: β-benzylation (according to the Melchiorre method) [16] with α-photooxygenation in a one pot procedure. Cinnamaldehyde (**12**) was reacted with benzyltrimethylsilane (**13**) in the presence of catalyst *cis*-**14** yielding aldehyde **1**. Once the reaction was completed, a solution of H_2_TPP and imidazolidinone **16** was added to the reaction mixture and oxygen was purged. After the reduction diol **6** was obtained in 12% yield, as a nearly equimolar mixture of *syn*-(2*R*,3*R*)- and *syn’*-(2*S*,3*S*)-**6** enantiomers (Table 2, entry 1). So, in the one-pot procedure a decrease in both yield and stereoselectivity was observed as compared to the two-step synthesis (Table 1, entry 2), suggesting that the reaction conditions are not compatible with one another.

**Table 2:** Photochemical difunctionalization of cinnamaldehyde (**12**) at the β and α-positions.

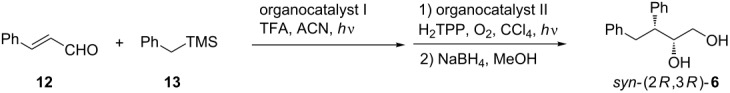							

Entry	Orgcat. I/II	Buffer [pH]	Yield [%]	dr *syn*:*anti*	er *syn*:*syn’*	er *anti*:*anti’*	Main stereoisomer

1	*cis*-**14**/**16**	–	12	>95:5	58:42	–	*syn* (2*R*,3*R*)
2	*cis*-**14**/**17**	6	28	80:20	55:45	86:14	*syn* (2*R*,3*R*)
3	*cis*-**14**/**17**	7	30	80:20	55:45	87:13	*syn* (2*R*,3*R*)

The use of silyl ether (*S*)-**17** instead of imidazolidinone **16** and phosphate buffer (pH 7) as an additive allowed to increase the yield of the ‘one-pot’ procedure to 30% (Table 2, entries 2 and 3). Additional experiments indicated that acetonitrile required in the first step has a negative effect on the subsequent α-photooxygenation reaction (for details see Supporting Information File 1).

### Determination of the absolute configuration of the product by electronic circular dichroism spectroscopy

**Scheme 5:**
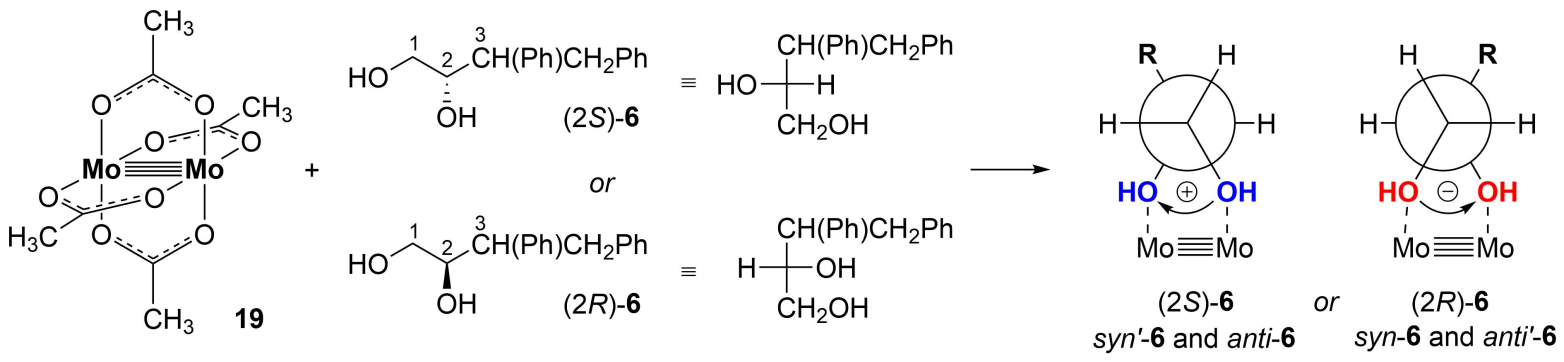
Schematic representation of the in situ methodology and preferred conformation of diols with Mo_2_ core.

Despite its rather simple structure diol **6** was not previously reported in the literature. The ratio of stereoisomers **6** was determined by HPLC analysis while the absolute configuration of the newly created stereocenter was established using a chiroptical spectroscopic method. Samples of stereoisomers *syn*-**6** and *anti’*-**6** obtained from the reaction with diarylprolinol silyl ether (*R*)-**18** (Table 1, entry 5) were analyzed using electronic circular dichroism spectroscopy (ECD) and TD-DFT methods. So-called in situ methodology with dimolybdenum tetraacetate (**19**) acting as auxiliary chromophores which proved a very useful tool in solving stereochemical problems [24–26], allowed to determine the absolute configuration at the C2 carbon atom of obtained diols (Scheme 5) [27–28].

**Figure 1:**
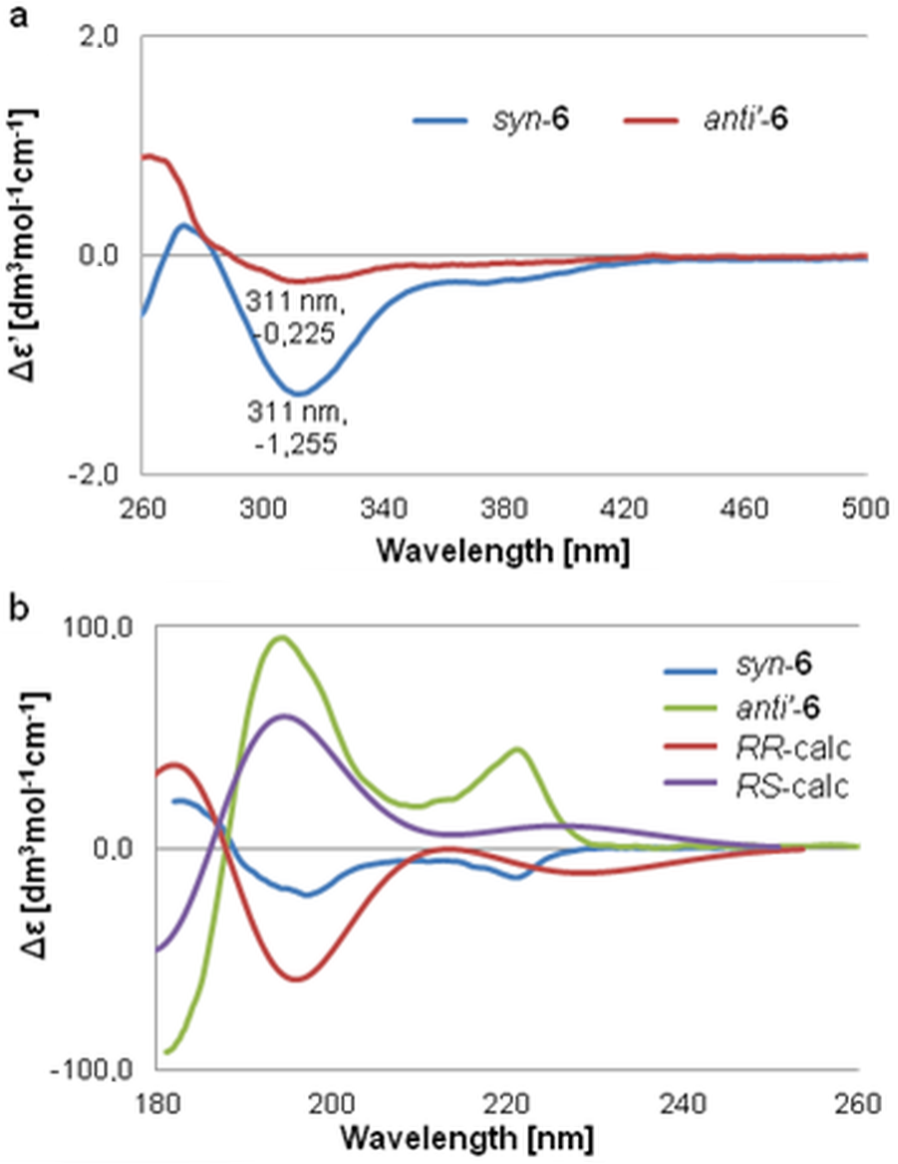
ECD spectra of diols *syn*-**6** and *anti’*-**6** recorded a) with **19** in DMSO and b) in acetonitrile compared with simulated ECD spectra.

In this method the achiral Mo complex **19** forms optically active complexes with chiral *vic*-diols allowing the application of ECD to compounds transparent in the UV–vis region. In solution, chirality of diols is transfered to the newly in situ-formed complexes. The signs of Cotton effects (CEs) observed in their spectra undergo the helicity rule linking the positive/negative sign of CE at about 300–400 nm with positive/negative sign of O–C–C–O torsion angles of the diol unit [27–29]. The negative sign of CE at 310 nm in the recorded spectra for both analyzed compounds *syn*-**6** and *anti’*-**6** correlates to the negative O–C–C–O torsion angle. Based on the preferred *gauche* conformation of the diol unit with large substituents (O–C–C–O fragments of molecule) in the antiperiplanar orientation as a result of steric repulsion, the absolute configuration at position 2 was assigned as (2*R*) in both compounds *syn*-**6** and *anti’*-**6** (Figure 1a).

The absolute configuration of the second stereocenter at C3 with the phenyl substituent was determined based on the ECD spectra recorded for pure *syn*-**6** and *anti’*-**6** diols (Figure 1b) [29]. The comparison of the experimental ECD curves with the calculated ones for two possible stereoisomers (2*R*,3*R*) and (2*R*,3*S*) allowed for unambiguous assignment of (2*R*,3*R*) absolute configuration to the *syn*-**6** isomer and (2*R*,3*S*) to the *anti’*-**6** isomer. Based on the known relationship between *syn*-**6** and *anti’*-**6** diols and the other two diastereoisomers we assigned their absolute configuration as (2*S*,3*S*) for *syn’*-**6** and (2*S*,3*R*) for *anti*-**6**, respectively.

### Asymmetric synthesis of 3,4-diphenylbutane-1,2-diol

**Scheme 6:**
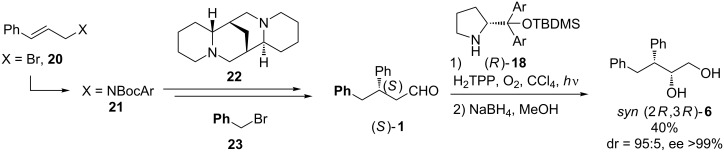
Asymmetric synthesis of 3,4-diphenylbutane-1,2-diol.

To confirm our conclusions concerning stereoselectivity in photooxygenation of chiral aldehydes, we synthesized enantiomerically pure aldehyde (*S*)-**1** from cinnamyl bromide (**20**) according to a literature procedure [30]. The reaction of (*S*)-3,4-diphenylbutanal (**1**) with singlet oxygen catalyzed by diarylprolinol silyl ether (*R*)-**18** furnished, after in situ reduction, diol (2*R*,3*R*)-**6** with high *syn*-diastereo- and enantioselectivity (Scheme 6).

## Conclusion

We have demonstrated that oxygenation of aldehydes with singlet oxygen can be successfully achieved in the presence of diphenylprolinol silyl ether affording diols in a highly diastereo- and enantioselective manner providing the presence of a substituent at the β-position. Using our procedure enantiopure (*S*)-3,4-diphenylbutanal ((*S*)-**1**) was transformed into (2*R*,3*R*)-3,4-diphenylbutane-1,2-diol in a highly stereoselective manner. This high level of stereoselectivity is rarely observed for reactions of singlet oxygen with substrates that do not possess chiral auxiliary or do not impose significant steric hindrance.

## Experimental

### General information

^1^H and ^13^C NMR spectra were recorded at rt on Bruker 400 and Varian 600 MHz instruments with TMS as an internal standard. The chemical shifts (δ) and coupling constants (*J*) are expressed in ppm and Hertz, respectively. High-resolution mass spectrometry (HRMS) data were obtained at the Synapt G2-S HDMS mass spectrometer equipped with an electrospray ion source and q-TOF type mass analyzer. Specific rotation was measured on a JASCO P-2000 polarimeter. The enantiomeric purity of diols was determined by chiral-phase HPLC analysis on Daicel Chiralpak ID (250 mm × 4.6 mm inside diameter) using a hexane/iPrOH mixture as a mobile phase. Thin-layer chromatography (TLC) was performed using Merck Silica Gel GF254, 0.20 mm thickness. All solvents and chemicals used in the syntheses were of reagent grade and were used without further purification. Aldehydes **1** [16,30], **2** [17], **3** [18] and organocatalysts **14** [31], **15** [32], **16** [33] were prepared by known procedures. Silyl ethers of diarylprolinols **17** and **18** were obtained from Sigma-Aldrich. Photochemical reactions were performed either using homemade photoreactors equipped with two LED warm white light bulbs (α-photooxygenation, scale >0.5 mmol), green high power LED (scale ≤0.5 mmol) or LED tapes (photochemical aldehydes synthesis), see Supporting Information File 1 for details.

### General procedure for α-photooxygenation

To a 10 mL vial a solution of *meso*-tetraphenylporphyrin (H_2_TPP, 0.4 mg, 0.63 µmol, 0.25 mol %) in CCl_4_ (1 mL) and NiPBA (17 μL, 0.1 mmol, 40 mol %) was added followed by aldehyde **1** (56 mg, 0.25 mmol), at 10 °C. The reaction mixture was stirred with gentle oxygen bubbling under irradiation (green high power LED) for 3 h. The light was turned off and a solution was transferred to a round bottom flask with MeOH (1 mL). The reaction mixture was then cooled to 0 °C before NaBH_4_ (50 mg, 1.3 mmol) was added. After stirring for 15 min at 0 °C the reaction was diluted with AcOEt, washed with a 1 N solution of HCl, and then saturated NaHCO_3_. The organic layer was dried over Na_2_SO_4_, filtered and concentrated. The crude mixture was purified by column chromatography (SiO_2_, hexanes/AcOEt), affording alcohol **9** (25 mg, 45%), diastereosomer *syn’*-**6** (14 mg, 23%), and diastereosomer *anti*-**6** (11 mg, 18%). Diastereosomer *syn’*-**6** was obtained as colorless oil, 14 mg, 23%. *R*_f_: 0.40 (hexanes/AcOEt 1:2); IR (film, *ν*_max_, cm^−1^): 3387, 3085, 3061, 3027, 2925, 1709, 1602, 1495, 1453, 1409, 1181, 1094, 1068, 1031, 745, 700, 562, 534; ^1^H NMR (400 MHz, CDCl_3_) δ_H_ 7.35–7.00 (m, 10H, ArH), 3.88 (dd, *J* = 9.1, 6.2 Hz, 1H, >C*H*OH), 3.65 (dd, *J* = 11.2, 3.2 Hz, 1H, -C*H*H-OH ), 3.42 (dd, *J* = 11.1, 7.4 Hz, 1H, -CH*H*-OH), 3.17 (dd, *J* = 12.5, 6.4 Hz, 1H, >CH-Ph), 2.99 (dd, *J* 12.8, 6.7 Hz, 1H, -C*H*H-Ph), 2.93 (dd, *J* = 12.4, 8.2 Hz, 1H, CH*H*-Ph), 1.88 (br s, 2H, 2 x OH); ^13^C NMR (100 MHz, CDCl_3_) δ_C_ 140.4, 139.9, 129.1, 128.9, 128.5, 128.2, 127.0, 126.0, 73.4, 65.3, 50.4, 38.6; HRESIMS *m*/*z*: [M + Na]^+^ calcd. for C_16_H_18_O_2_Na, 265.1204; found, 265.1199. Diastereoisomer *anti*-**6** obtained as colorless oil, 11 mg, 18%. *R*_f_: 0,33 (hexanes/AcOEt 1:2); IR (film, *ν*_max_, cm^−1^): 3375, 3085, 3061, 3028, 2925, 1706, 1602, 1495, 1452, 1097, 1070, 1048, 1030, 876, 761, 700, 626, 556; ^1^H NMR (400 MHz, CDCl_3_) δ_H_ 7.25–7.08 (m, 6H, ArH), 7.04 (d, *J* = 6.7 Hz, 2H, ArH), 6.96 (d, *J* = 6.7 Hz, 2H, ArH), 3.98–3.90 (m, 1H, >C*H*OH), 3.46–3.28 (m, 3H, -CH_2_OH, >CH-Ph), 2.92–2.90 (m, 2H, -CH_2_Ph), 2.38 (br s, 1H, OH) 1.56 (br s, 1H, OH); ^13^C NMR (100 MHz, CDCl_3_) δ_C_ 140.8, 140.0, 129.1, 128.4, 128.3, 128.0, 126.7, 125.8, 75.3, 65.0, 51.4, 38.7; HRESIMS *m*/*z*: [M + Na]^+^ calcd for C_16_H_18_O_2_Na, 265.1204; found, 265,1198. HPLC analysis on Daicel Chiralpak ID (250 mm × 4.6 mm inside diameter) using a hexane/AcOEt, 80:20 (v/v) as a mobile phase, with the flow rate set at 1.5 mL/min. The retention times were 7.3; 7.9; 9.4 and 13.4 min for (2*S*,3*R*), (2*R*,3*S*), (2*R*,3*R*) and (2*S*,3*S*), respectively. Alcohol **9** was obtained as a colorless oil, 25 mg, 45%. ^1^H NMR (400 MHz, CDCl_3_) δ_H_ 7.30–7.25 (m, 2H, ArH), 7.22–7.10 (m, 6H, ArH), 7.06–7.04 (m, 2H, ArH), 3.52–3.50 (m, 1H, >CH-), 3.43–3.40 (m, 1H, -C*H*H-Ar), 2.99–2,90 (m, 1H, -CH*H*-Ar), 2.91–2.88 (m, 2H, CH_2_OH) 1.98–1.93 (m, 1H, C*H*HCH_2_OH) 1.86–1.80 (m, 1H, CH*H*CH_2_OH), 1.14 (br s, 1H, OH). Analytical data corresponds to the literature data [34].

### General procedure for α-photooxygenation with chiral organocatalysts

Reactions with chiral organocatalysts **15**–**18** reported in Table 1 were performed according to the procedure described above but only 20 mol %, 0.05 mmol of catalyst were used. Reactions catalyzed by diaryl prolinols (*S*)-**18** and (*R*)-**18** yielded *anti*-**6** and *syn*-**6**, respectively. Enantiomer *S*,*R* (*anti-***6**) 82% ee [α]_D_^20^ −147.0 (*c* 0.6, CHCl_3_). Enantiomer *R*,*R* (*syn*-**6**) 99% ee, [α]_D_^20^ −104.0 (*c* 0.4, CHCl_3_).

### General procedure for α-photooxygenation with phosphate buffer solution

To a 10 mL vial a solution of *meso*-tetraphenylporphyrin (H_2_TPP, 0.4 mg, 0.63 µmol, 0.25 mol %) in CCl_4_ (1 mL) and organocatalyst (*S*)-**17** (18 mg, 0.05 mmol, 20 mol %) were added followed by a phosphate buffer solution (0.5 mL, 0.1 M, pH 6.0 or 7.0). After cooling to 10 °C, aldehyde **1** (56 mg, 0.25 mmol) was added to the two-phase reaction mixture, which was then extensively stirred for 3 h with oxygen bubbling under irradiation (green high power LED). The light was turned off and the mixture was left for phase separation. The organic layer was transferred to a round bottom flask and mixed with MeOH (1 mL). The reaction mixture was then cooled to 0 °C before NaBH_4_ (50 mg, 1.3 mmol) was added. After stirring for 15 min at 0 °C the reaction mixture was diluted with AcOEt, washed with 1 N solution of HCl, and then saturated NaHCO_3_. The organic layer was dried over Na_2_SO_4_, filtered and concentrated. The crude mixture was purified by column chromatography (SiO_2_, hexanes/AcOEt), affording (with pH 6.0 buffer) diastereosomer *syn’*-**6** (2*S*,3*S-***6**, 14 mg, 23%), diastereosomer *anti*-**6** (2*S*,3*R-***6**, 22 mg, 36%), respectively. The relative ratio of stereoisomers was determined by HPLC analysis: Daicel Chiralpak ID (250 mm × 4.6 mm), hexane/AcOEt, 80:20 (v/v) flow rate 1.5 mL/min. The retention times were 7.3; 7.9; 9.4 and 13.4 min for (2*S*,3*R*), (2*R*,3*S*), (2*R*,3*R*) and (2*S*,3*S*), respectively.

### One-pot two-step procedure

To a 10 mL vial filled with argon a solution of imidazolidinone *cis*-**14** (25 mg, 0.1 mmol, 20 mol %) in acetonitrile (1 mL), TFA (15 µL, 0.2 mmol, 40 mol %), benzyltrimethylsilane (**13**, 95 µL, 0.5 mmol) and cinnamaldehyde (**12**, 190 µL, 1.5 mmol) were added. The resulting solution was purged with argon for 5 min before irradiation (violet high power LED), at rt, started. After 24 h the light was turned off and the solution of *meso*-tetraphenylporphyrin (H_2_TPP, 0.8 mg, 1.25 µmol, 0.5 mol %), catalyst (*S*)-**17** (37 mg, 0.1 mmol, 20 mol %) in CCl_4_ (2 mL) and phosphate buffer solution (1 mL, 0.1 M, pH 6.0 or 7.0) were added to the reaction mixture. The solution was then stirred and irradiated (green single LED) for 3 h with gentle oxygen bubbling. The light was turned off and the mixture was left for phase separation. The organic layer was transferred to a round bottom flask and mixed with MeOH (2 mL). The reaction mixture was then cooled to 0 °C before NaBH_4_ (100 mg, 2.6 mmol) was added. After stirring for 15 min at 0 °C the reaction mixture was diluted with AcOEt, washed with 1 N solution of HCl and saturated NaHCO_3_. The organic layer was dried over Na_2_SO_4_, filtered and concentrated. The crude product was purified by column chromatography (SiO_2_, hexanes/AcOEt, 80:20) affording 36 mg (30%, pH 7.0) or 34 mg (28%, pH 6.0) of the desired product **6**.

### Electronic circular dichroism

The ECD spectra of free diols were collected at room temperature in acetonitrile (for UV spectroscopy, Fluka) on a Jasco J-715 spectropolarimeter at 0.2 nm/step with an integration time of 0.5 s over the range 180–400 nm with 100 nm/min scan speed, 5 scans. For the in situ ECD standard measurements the chiral *vic*-diol (1–8 mg, ca. 0.003 M/L) was dissolved in a stock solution of the [Mo_2_(O_2_CCH_3_)_4_] (4–6 mg, ca. 0.002 M/L) in DMSO (5 mL) (for UV spectroscopy, Fluka) so that the molar ratio of the stock complex to ligand was about 1.5:1, in general. The measurements were performed on a Jasco J-715 spectropolarimeter with parameters: 0.5 nm/step with an integration time of 0.25 s over the range 248–700 nm with 200 nm/min scan speed, 5 scans. Since the real complex structure as well as the concentration of the chiral complex formed in solution was not known, the CD data are presented as the Δε’ values. These Δε’ values are calculated in the usual way as Δε’ = Δ*A*/*c* × *d*, where *c* is the molar concentration of the chiral ligand, assuming 100% complexation (*A* = absorption; *d* = path length of the cell). Δε’ is expressed in [M^−1^ cm^−1^] units.

### Conformational analysis and ECD calculations

The conformational search was performed with ComputeVOA [35] using the MMFF94 force field within 5 kcal/mol energy ranges. Further optimization was carried out at DFT level using the B3LYP functional and the Def2TZVP basis set in the Gaussian 09 [36]. Simulations of ECD spectra were carried out with TD-DFT methods for conformers found in the range of 2.5 kcal/mol. The B3LYP functional in conjunction with the Def2TZVP basis set was used for computing the first 80 electronic transitions. The final spectrum was obtained by Boltzmann averaging (*T* = 298 K) according to the population percentages of individual conformers based on the relative Gibbs energies calculated at the same level of theory.

## Supporting Information

File 1 Photochemical equipment, experiments for the optimization of the one-pot procedure, analytical data for **7**, **8**, **10**, **11**, HPLC chromatograms, and NMR spectra.
