# Supplementary material for: α-Photooxygenation of chiral aldehydes with singlet oxygen
Source: Beilstein J Org Chem. 2019 Aug 30;15:2076–84. doi: 10.3762/bjoc.15.205 (PMC6720656; doi:10.3762/bjoc.15.205)
Supplement: File 1 — Photochemical equipment, experiments for the optimization of the one-pot procedure, analytical data for 7, 8, 10, 11, HPLC chromatograms, and NMR spectra. [file Beilstein_J_Org_Chem-15-2076-s001.pdf]

BEILSTEIN JOURNAL OF ORGANIC CHEMISTRY

Supporting Information

for

α-Photooxygenation of chiral aldehydes with singlet oxygen

Dominika J. Walaszek, Magdalena Jawiczuk, Jakub Durka, Olga Drapała and Dorota Gryko

Beilstein J. Org. Chem. **2019,** *15,* 2076–2084. doi:10.3762/bjoc.15.205

Photochemical equipment, experiments for the optimization of the one-pot procedure, analytical data for 7, 8, 10, 11, HPLC chromatograms, and NMR spectra

License and Terms: This is a supporting information file under the terms of the Creative Commons Attribution License (http://creativecommons.org/licenses/by/4.0). Please note that the reuse, redistribution and reproduction in particular requires that the authors and source are credited.
The license is subject to the *Beilstein Journal of Organic Chemistry* terms and conditions: (https://www.beilstein-journals.org/bjoc)

Table of Content

1. Photochemical reactions – equipment S2

2. Photooxygenation reaction – experimental procedures S3

   a. Diol **7** (4-(1,2-dihydroxyoctan-3-yl)benzonitrile) - *photooxygenation of aldehyde 2 (4-(1-oxooctan-3-yl)benzonitrile)* S3

   b. Diol **8** (benzyl 4-(1,2-dihydroxyethyl)-2-phenylnonanoate) - *photooxygenation of aldehyde **3** (benzyl 4-(2-oxoethyl)-2-phenylnonanoate)* S4

3. Additional experiments conducted in order to find the cause of decrease in efficiency of the 'one-pot' process S5

4. HPLC chromarograms S6

5. NMR S7

6. References S19

1. Photochemical reactions – equipment

Photochemical reactions were performed in homemade photoreactors with LED bulbs, LED tapes or high power single LED depending on the scale and the colour of light required.

Photooxygenation reactions performed on a scale larger than 0.5 mmol were conducted in a PCV box with two warm white LED bulbs. To assure constant flow of oxygen, effective cooling, and mixing, a three-neck round-bottom flask was equipped with a 'cold finger', capillary and outer joint (Figure S1a).

Aldehyde **1** was obtained according to the Melchiorre method [S1] in a violet LED stripes photoreactor or in reactor with eight violet 1 W LEDs (Figure S1b or c) depending on the scale.

Aldehydes **2** and **3** were obtained according to the MacMillan methods [S2,S3] using violet or blue LED tape photoreactors, respectively (Figure S1b).

Small-scale photooxygenation reactions were conducted in vials in a photoreactor with green high power LED. A coolant circulated inside the aluminium block allowed to control the reaction temperature, while constant flow of the oxygen was provided by teflon capillaries (Figure S1d).

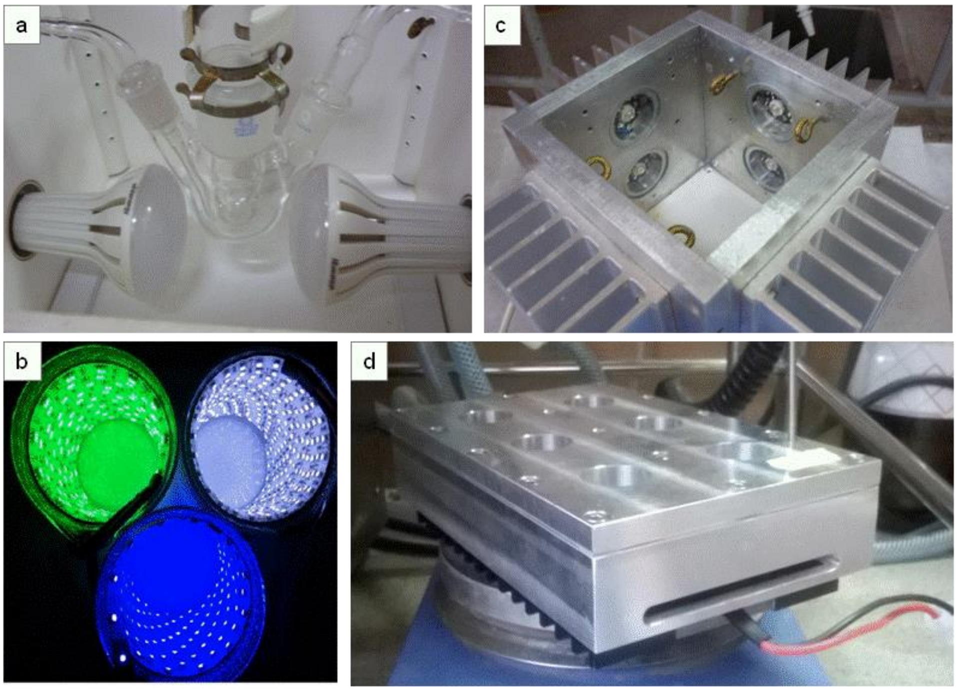

Figure S1.

2. Photooxygenation reactions – experimental procedures

a. Diol **7** (4-(1,2-dihydroxyoctan-3-yl)benzonitrile) - *photooxygenation of aldehyde* ***2*** *(4-(1-oxooctan-3-yl)benzonitrile)*

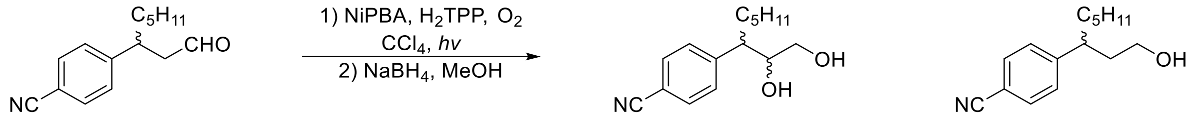

2 **7**, 34% **10**, 54%

To a solution of *meso*-tetraphenylporphyrin (H_2TPP, 1.5 mg, 2.5 µmol, 0.25 mol%) in CCl_4 (4 mL) NiPBA (66 µL, 0.4 mmol, 40 mol %) and aldehyde **2** (230 mg, 1.0 mmol) were added at 10 °C. The reaction mixture was stirred at this temperature with gentle oxygen bubbling under irradiation (2 white LED bulbs) for 3 h. The light was turned off and MeOH (4 mL) was added to a solution. The reaction mixture was then cooled to 0 °C before $NaBH_4$ (200 mg, 5.3 mmol) was added. After stirring for 15 min at 0 °C the reaction mixture was diluted with AcOEt, washed with 1 N solution of HCl, saturated $NaHCO_3$. The organic layer was dried over Na_2SO_4, filtered and concentrated. Crude products were purified by column chromatography (SiO_2, hexanes/AcOEt, for alcohol **10** 80:20, for desired product **7** 60:40). Diol **7** (colourless oil) obtained as an inseparable mixture of two diasteroisomers (approx. 1.4:1), 85 mg, 34%. **R_f**: 0,40 (hexanes/AcOEt, 1:2). **IR** (film, *v*max, cm^{-1}): 3409, 2954, 2929, 2859, 2228, 1606, 1503, 1465, 1415, 1379, 1202, 1177, 1086, 1035, 876, 734, 601, 571. **^{1}H NMR** (500 MHz, $CDCl_3$): δ_H 7.60 (d, *J* 8.2 Hz, 2H, ArH, major), 7.59 (d, *J* 8.2 Hz, 2H, ArH, minor), 7.35 (d, *J* 8.3 Hz, 2H, ArH, major), 7.28 (d, *J* 8.3 Hz, 2H, ArH, minor), 3.94 – 3.84 (m, 1H, >CHOH, major), 3.76 (ddd, *J* 8.3, 7.4, 2.9 Hz, 1H, >CHOH, minor), 3.68 (dd, *J* 11.1, 3.2 Hz, 1H, -CH*H*-OH, major), 3.44 – 3.34 (m, 2H, 2x -C*H*H-OH, major + minor), 3.30 – 3.20 (dd, *J* 11.0, 7.5 1H, -CH*H*-OH, minor), 2.81 – 2.67 (m, 2H, 2x >CH-Ar, major + minor), 2.29 (br s, 4H, 4x OH), 2.05 – 1.58 (m, 4H, 2x -C*H*$_2$-C_4H_9, major + minor), 1.30 – 0.95 (m, 12H, 2x -$(CH_2)_3$-, major + minor), 0.85 – 0.80 (m, 6H, 2x CH_3, major + minor). **^{13}C NMR** (125 MHz, $CDCl_3$): δ_C 147.9, 147.5, 132.3, 132.1, 129.6, 129.0, 118.9, 118.8, 110.4, 110.3, 75.4, 74.6, 65.0, 64.8, 49.3, 48.8, 31.8, 31.7, 31.6, 31.3, 26.9, 26.8, 22.4, 22.3, 13.9. **HRMS** (ESI) calcd for $C_{15}H_{21}NO_2Na$ $[M + Na]^+$ 270,1470. Found: 270,1463. **Anal.** calcd for $C_{15}H_{21}NO_2$: C, 72.84; H, 8.56; N, 5.66. Found: C, 72.70; H, 8.54; N, 5.61. Alcohol **10** obtained as colorless oil, 125 mg, 54%. **R_f**: 0,75 (hexanes/AcOEt, 1:2). **IR** (film, *v*max, cm^{-1}): 3424, 2954, 2929, 2857, 2228, 1606, 1503, 1465, 1415, 1378, 1302, 1178, 1103, 1049, 836, 575. **^{1}H NMR** (400 MHz, $CDCl_3$): δ_H 7,56 (d, *J* = 8,3 Hz, 2H, ArH), 7,26 (d, *J* = 8,3 Hz, 2H, ArH), 3,57 – 3,46 (m, 1H, -CH*H*-OH), 3,44 – 3,32 (m, 1H, -C*H*H-OH), 2,86 – 2,69 (m, 1H, >CH-Ar), 1,96 – 1,90 (m, 1H, -CH*H*-CH_2OH), 1,80 – 1,70 (m, 1H, -C*H*H-CH_2OH), 1,64 – 1,54 (m, 2H, -C*H*$_2$-C_4H_9), 1,4 (br s, 1H, OH), 1,30 – 1,05 (m, 6H, -$(CH_2)_3$-), 0,8 (t, *J* = 8,0 Hz, 3H, CH_3). **^{13}C NMR** (100 MHz, $CDCl_3$): δ_C 151.3, 132.2, 128.5, 119.0, 109.9, 60.5, 42.4, 39.2, 36.4, 31.7, 27.0, 22.4, 13.9. **HRMS** (ESI) calcd for $C_{15}H_{21}NONa$ $[M + Na]^+$ 254,1521. Found: 254,1510. **Anal.** calcd for $C_{15}H_{21}NO$: C, 77.88; H, 9.15; N, 6.05. Found: C, 77.74; H, 9.39; N, 6.06.

b. Diol **8** (benzyl 4-(1,2-dihydroxyethyl)-2-phenylnonanoate) - *photooxygenation of aldehyde **3** (benzyl 4-(2-oxoethyl)-2-phenylnonanoate)*

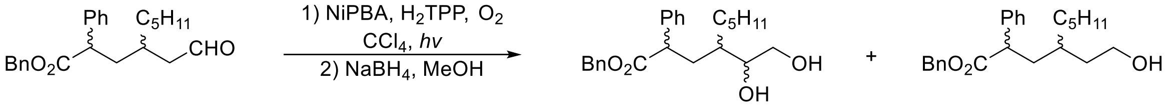

3 **8**, 31% **11**, 22%

Diol **8** was obtained according to the procedure described for aldehyde **2** with *meso*-tetraphenylporphyrin (H_2TPP, 1.5 mg, 2.5 µmol, 0.25 mol %) in CCl_4 (4 mL), NiPBA (66 µL, 0.4 mmol, 40 mol %) and aldehyde **3** (336 mg, 1.0 mmol). Alcohol **11** and desired product **8** were purified by column chromatography (SiO_2, hexane/AcOEt, respectively 80:20 and 60:40). Diol **8** (colourless oil) obtained as an inseparable mixture of four diasteroisomers, 120 mg, 31%. **R_f**: 0,45 (hexanes/AcOEt, 1:2). **IR** (film, *v*max, cm^{-1}): 3408, 2954, 2929, 2859, 1734, 1496, 1454, 1379, 1336, 1267, 1214, 1158, 1065, 1030, 1005, 733, 698. **^{1}H NMR** (600 MHz, $CDCl_3$): δ_H 7.33 – 7.18 (m, 10H, ArH), 5.15 – 5.10 (m, 1H, ArCH*H*-CO_2), 5.08 – 5.02 (m, 1H, ArC*H*H-CO_2), 3.80 – 3.42 (m, 4H, -C*H*$_2$OH, >C*H*OH, ArCH<), 2.18 – 1.65 (m, 4H, >C*H*-C_5H_{11}, -C*H*$_2$-CHAr, OH), 1.38 – 1.16 (m, 9H, -$(CH_2)_4$-, OH), 0.86 (t, 3H, CH_3). **^{13}C NMR** (150 MHz, $CDCl_3$): δ_C = 174.4, 173.8, 139.1, 138.8, 135.9, 135.7, 128.71, 128.67, 128.5, 128.4, 128.04, 127.99, 127.97, 127.8, 127.3, 73.74, 73.72, 73.56, 73.55, 66.7, 66.66, 66.5, 64.9, 64.5, 64.4, 50.1, 49.9, 49.5, 49.1, 39.2, 38.5, 34.0, 33.9, 32.2, 32.1, 29.8, 29.4, 26.7, 26.6, 22.5, 14.1, 14.0. **HRMS** (ESI) calcd for $C_{24}H_{32}O_4Na$ $[M + Na]^+$ 407.2198. Found: 407.2193. **Anal.** calcd for $C_{24}H_{32}O_4$: C, 74.97; H, 8.39. Found: C, 75.09; H, 8.35. Alcohol **11** obtained as colorless oil, 80 mg, 22% **R_f**: 0,80 (hexanes/AcOEt, 1:2). **IR** (film, *v*max, cm^{-1}): 3421, 3063, 3032, 2953, 2927, 2858, 1735, 1496, 1454, 1379, 1335, 1274, 1212, 1155, 1054, 1031, 1003, 733, 698. **^{1}H NMR** (400 MHz, $CDCl_3$): δ_H 7.36 – 7.20 (m, 10H, ArH), 5.13 – 5.10 (m, 1H, ArCH*H*-CO_2), 5.09 – 5.05 (m, 1H, ArC*H*H-CO_2), 3.76 – 3.65 (m, 2H, -C*H*$_2$OH), 3.64 – 3.55 (m, 1H, ArCH<), 2.18 – 2.00 (m, 1H, >C*H*-C_5H_{11}), 1.80 – 1.60 (m, 2H, -CH_2-), 1.55 – 1.40 (m, 2H, -CH_2-), 1.35 – 1.15 (m, 9H, -$(CH_2)_4$-, OH), 0.86 (2x t, 3H, CH_3). **^{13}C NMR** (100 MHz, $CDCl_3$): δ_C 174.2, 173.9, 139.3, 139.1, 135.9, 135.88, 128.6, 128.4, 128.1, 128.0, 127.99, 127.98, 127.9 127.3, 127.2, 66.5, 66.4, 60.8, 60.7, 49.5, 49.4, 37.7, 37.67, 36.62, 33.9, 32.5, 32.3, 32.1, 32.06, 25.9, 25.8, 22.6, 14.04, 14.02. **HRMS** (ESI) calcd for $C_{24}H_{32}O_3Na$ $[M + Na]^+$ 391,2249. Found: 391.2249. **Anal.** calcd for $C_{24}H_{32}O_3$: C, 78.22; H, 8.75. Found: C, 77.98; H, 8.84.

3. Additional experiments conducted in order to find the cause of decrease in efficiency of the 'one-pot' process

In a 'one-pot' β-benzylation and α-oxygenation process, both yields and selectivities were lower than that observed in the two-step experiment. In this section we describe experiments performed in order to find out which reagents or solvents present in the reaction mixture after the first step have a negative effect on subsequent photooxygenation (Table S1, entries 1–3). These results were compared with the analogous α-photooxygenation with the most efficient catalyst – ether **17** preformed in absence and presence of buffer (Table S1, entry 4 and 5, also Table 1, entry 3 in the article).

Table S1. Parameters influencing the yield and stereoselectivity of the α-photooxygenation of 3,4-diphenylbutanal (**1**).

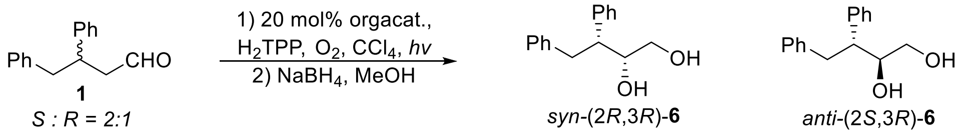

Entry	Cat.	Solvent(s) [buffer pH]	Yield [%]	dr *syn:anti*	er *syn:syn'*	er *anti:anti'*	Main stereoisomer
1	*cis*-**14**/**17**	CCl_4	44	1:2	22:78	85:15	*anti* (2*S*,3*R*)
2	(*S*)-**17**	ACN/CCl_4/[7]	25	4:1	55:45	70:30	*syn* (2*R*,3*R*)
3	(*S*)-**17**	ACN	13	1:1	45:55	40:60	*syn* (2*R*,3*R*)
4	(*S*)-**17**	CCl_4	52	1:2	19:81	91:9	*anti* (2*S*,3*R*)
5	(*S*)-**17**	CCl_4/[7]	59	1:2	17:83	95:5	*anti* (2*S*,3*R*)

α-Photooxygenation in the presence of catalysts *cis*-**14** and **17** gave similar results to the one obtained in the reaction performed under optimal conditions (compare entries 1 and 4) indicating that imidazolidinone *cis*-**14** did not interfere significantly in the investigated process. Both the efficiency and *syn*-stereoselectivity decreased when the reaction was performed in a mixture of ACN, CCl_4 and a buffer (entry 2). The reaction in polar aprotic ACN provided desired diol **6** in low yield as an equimolar mixture of all diastreoisomers, undoubtedly confirming the negative impact of this solvent on the α-photooxygenation process (entry 3).

4. HPLC chromatograms

HPLC analysis were performed on Daicel Chiralpak ID (250 mm × 4.6 mm inside diameter) with hexane/AcOEt, 80:20 (v/v) as a mobile phase, with the flow rate set at 1.5 mL/min. The retention times were 7.3; 7.9; 9.4 and 13.4 min for (2*S*,3*R*), (2*R*,3*S*), (2*R*,3*R*) and (2*S*,3*S*), respectively.

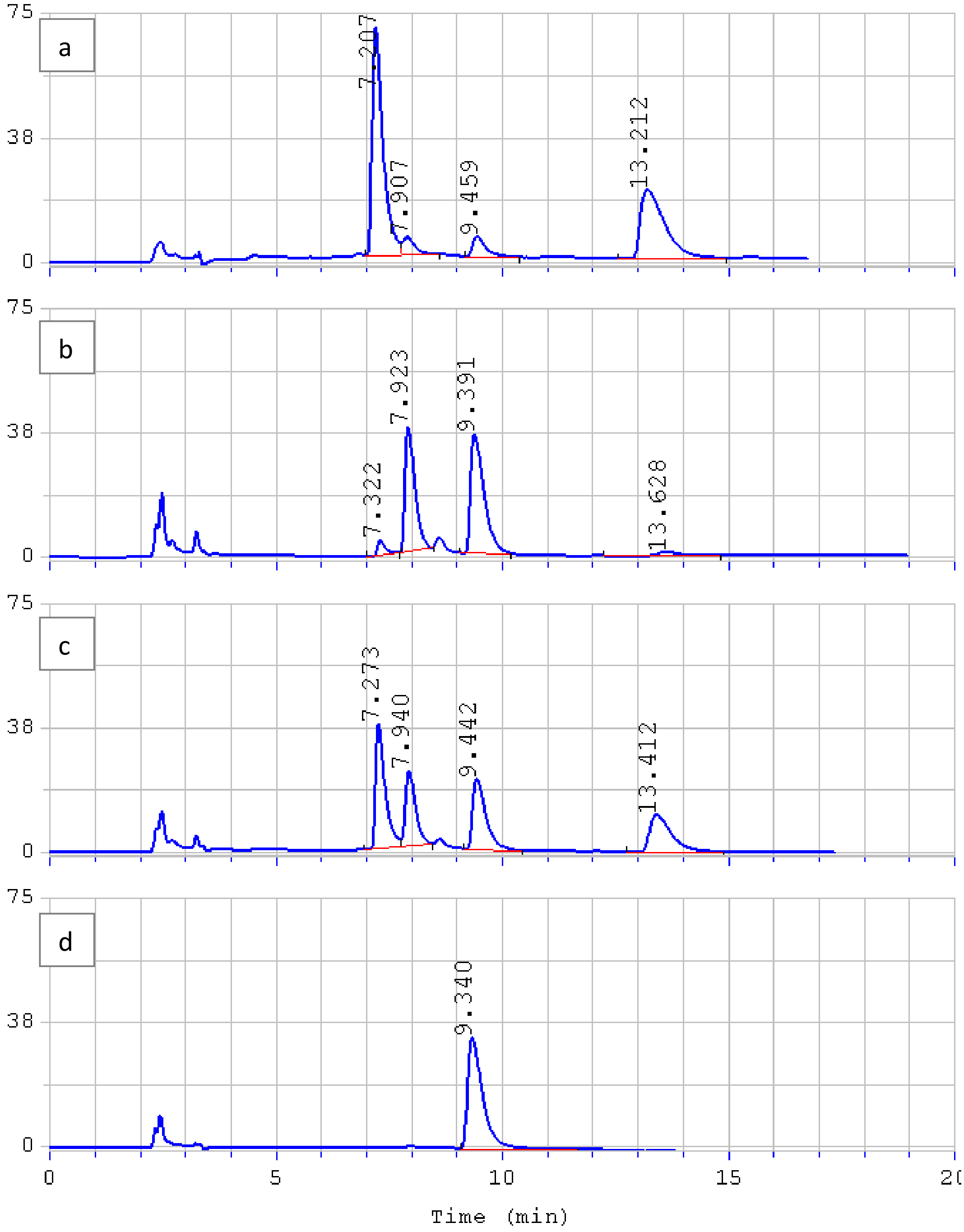

Figure S2. Chromatograms of 3,4-diphenylbutane-1,2-diol (**6**) samples obtained in the reaction with a) (*S*)-**18**, b) (*R*)-**18**, c) NiPBA d) with (*R*)-**18**, starting from enantiopure (*S*)-3,4-diphenylbutanal (**1**).

5. NMR

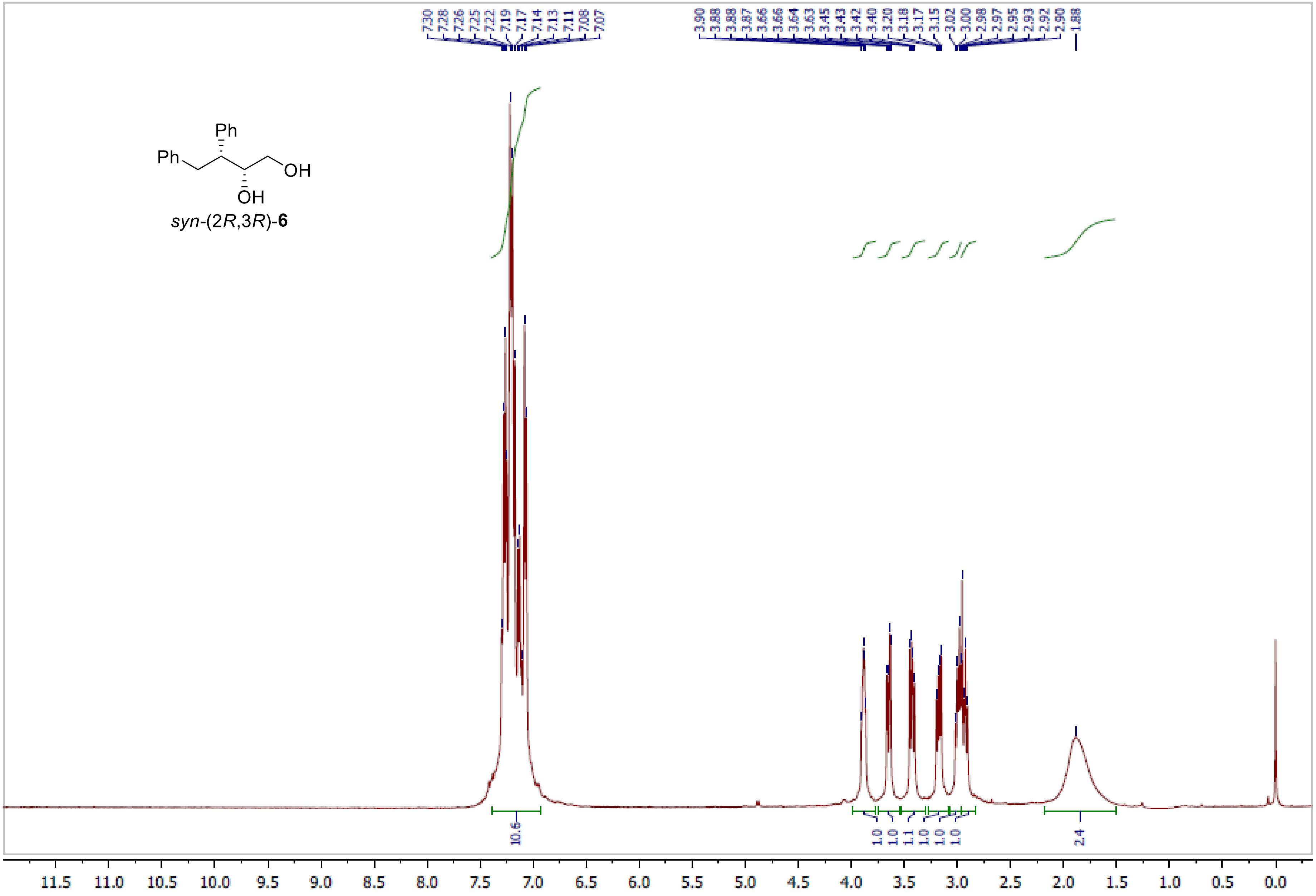
7.30
7.28
7.26
7.25
7.22
7.19
7.17
7.14
7.13
7.11
7.08
7.07
3.90
3.88
3.88
3.87
3.66
3.66
3.64
3.63
3.45
3.43
3.42
3.40
3.20
3.18
3.17
3.15
3.02
3.00
2.98
2.97
2.95
2.93
2.92
2.90
1.88
Ph
Ph
OH
OH
syn-(2R,3R)-6
10.6
1.0
1.0
1.1
1.0
1.0
1.0
2.4
11.5 11.0 10.5 10.0 9.5 9.0 8.5 8.0 7.5 7.0 6.5 6.0 5.5 5.0 4.5 4.0 3.5 3.0 2.5 2.0 1.5 1.0 0.5 0.0

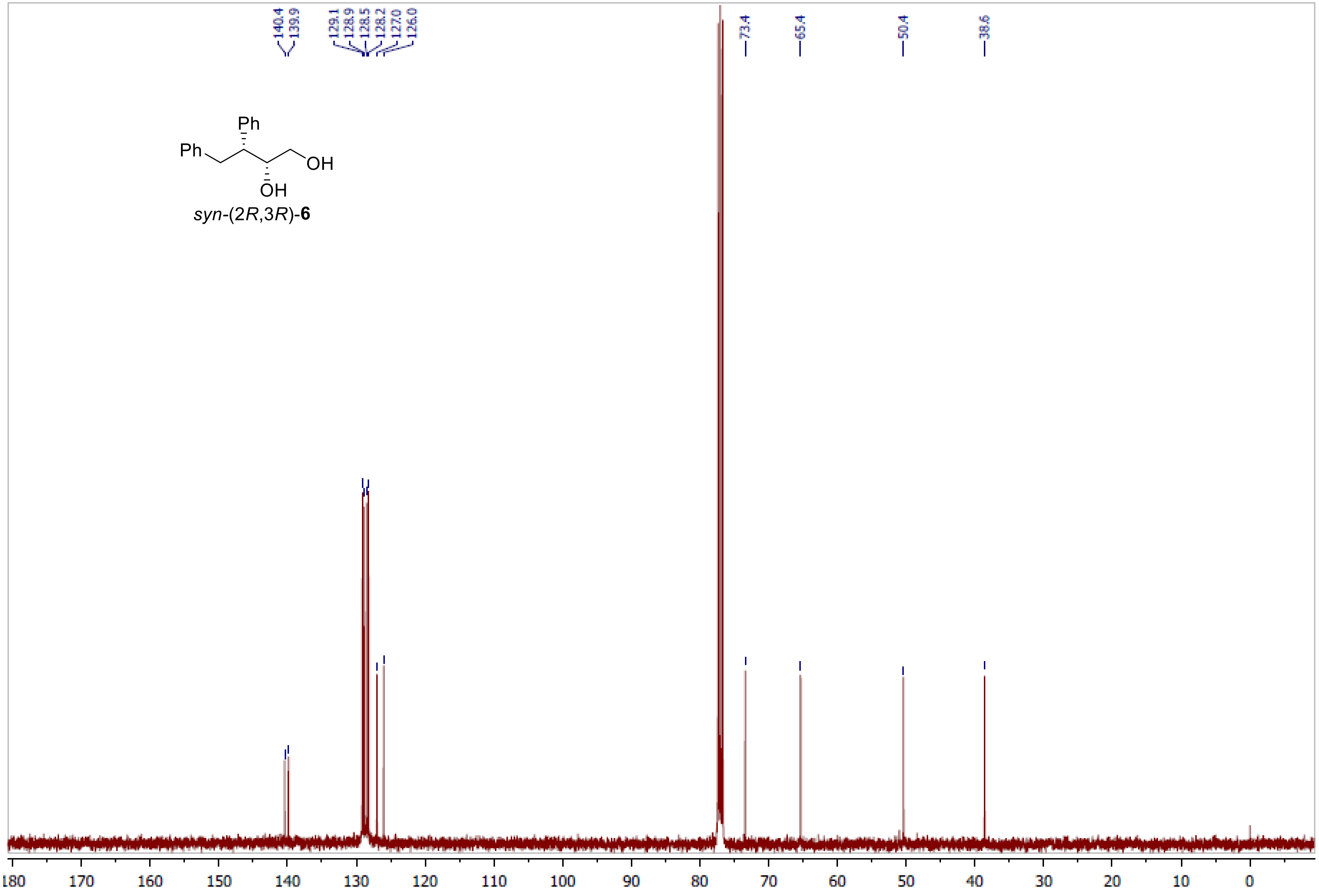
140.4
139.9
129.1
128.9
128.5
128.2
127.0
126.0
73.4
65.4
50.4
38.6
Ph
Ph
OH
OH
syn-(2R,3R)-6
180
170
160
150
140
130
120
110
100
90
80
70
60
50
40
30
20
10
0

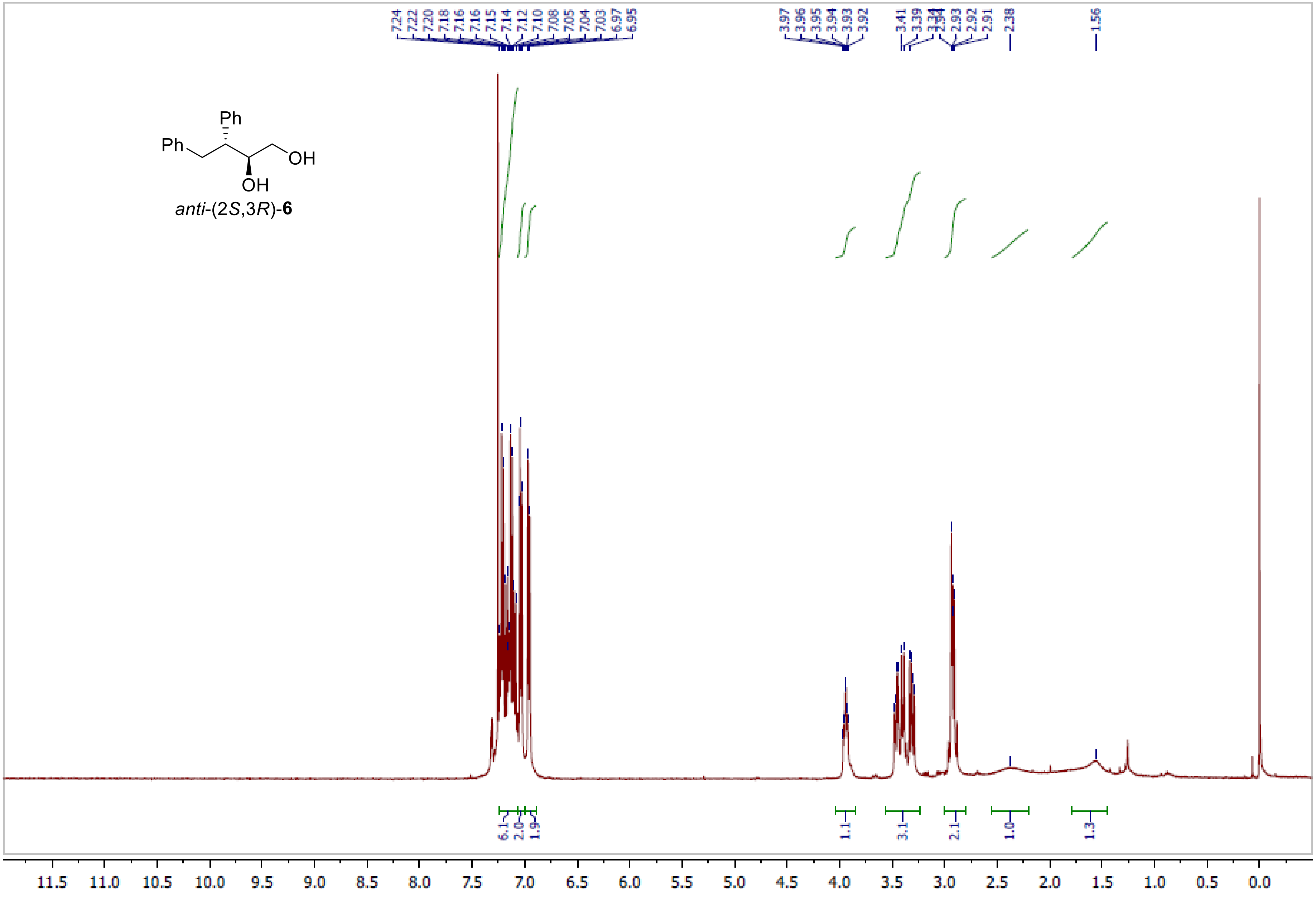

Ph
Ph
OH
OH
anti-(2S,3R)-6
7.24
7.22
7.20
7.18
7.16
7.16
7.15
7.14
7.12
7.10
7.08
7.05
7.04
7.03
6.97
6.95
3.97
3.96
3.95
3.94
3.93
3.92
3.41
3.39
2.93
2.92
2.91
2.38
1.56
6.1
2.0
1.9
1.1
3.1
2.1
1.0
1.3
11.5
11.0
10.5
10.0
9.5
9.0
8.5
8.0
7.5
7.0
6.5
6.0
5.5
5.0
4.5
4.0
3.5
3.0
2.5
2.0
1.5
1.0
0.5
0.0

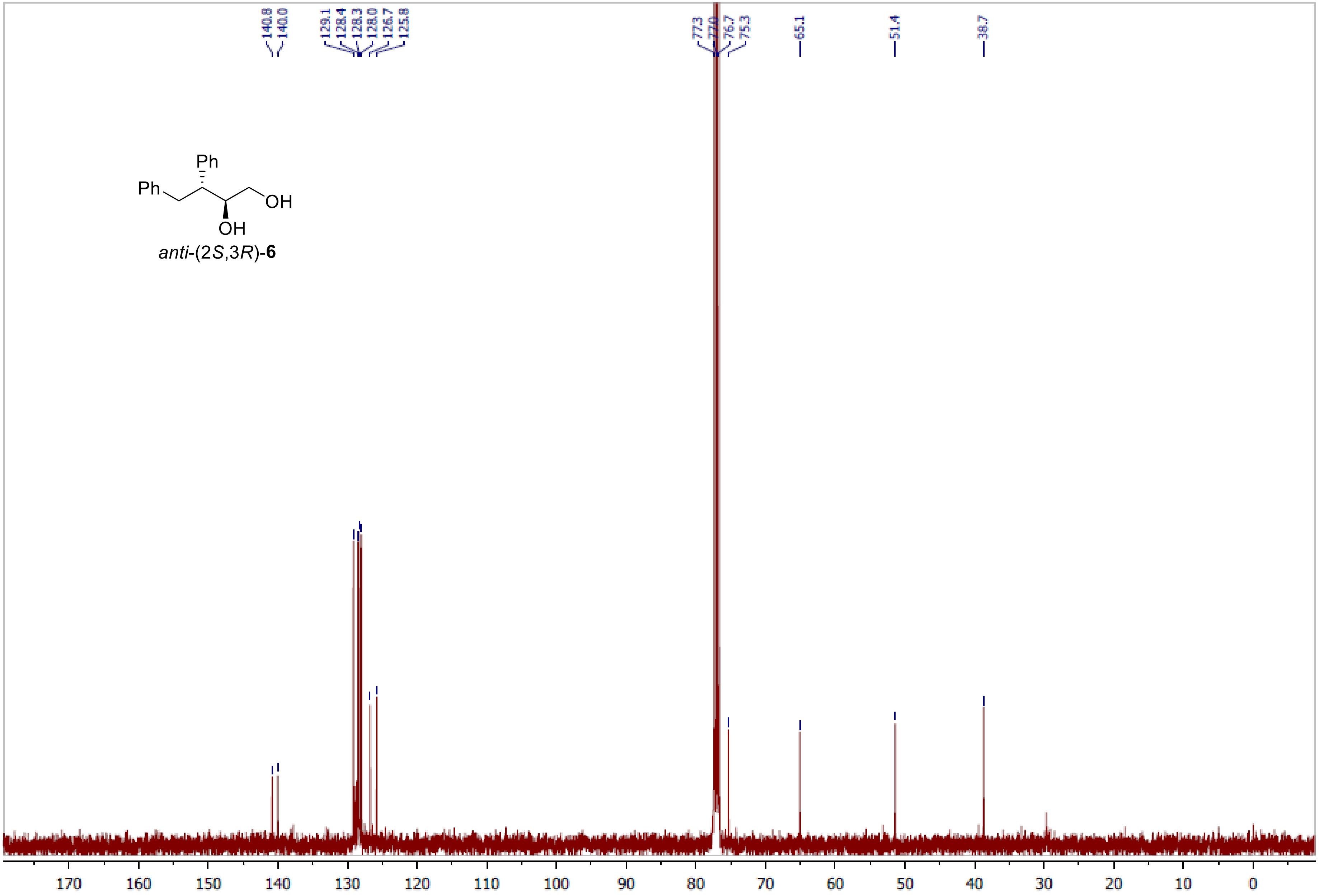
140.8
140.0
129.1
128.4
128.3
128.0
126.7
125.8
77.3
77.0
76.7
75.3
65.1
51.4
38.7
Ph
Ph
OH
OH
170 160 150 140 130 120 110 100 90 80 70 60 50 40 30 20 10 0

anti-(2*S*,3*R*)-**6**

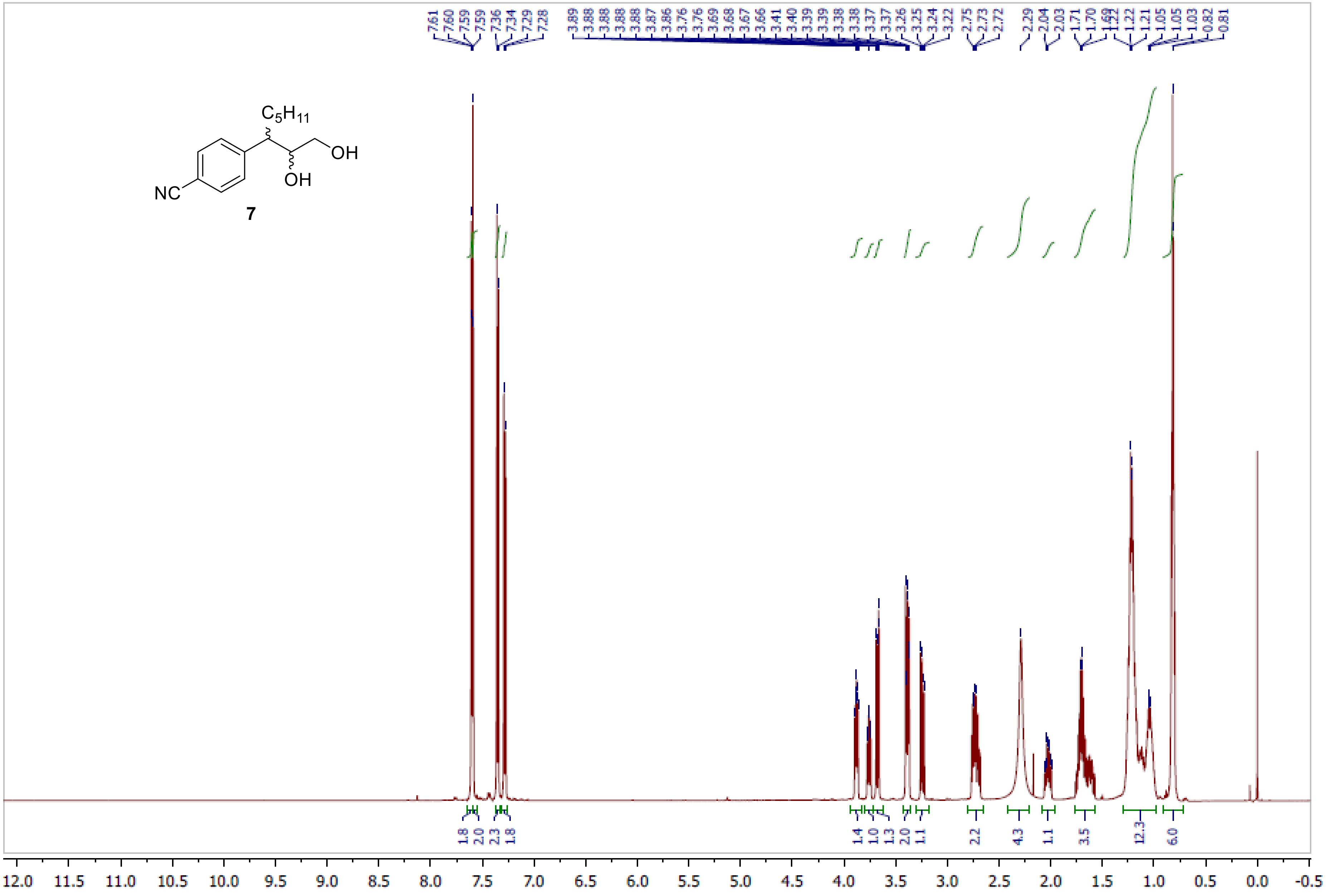
C5H11
OH
OH
NC
7
7.61
7.60
7.59
7.59
7.36
7.34
7.29
7.28
3.89
3.88
3.88
3.88
3.88
3.87
3.86
3.76
3.76
3.69
3.68
3.67
3.66
3.41
3.40
3.39
3.39
3.38
3.38
3.37
3.37
3.26
3.25
3.24
3.22
2.75
2.73
2.72
2.29
2.04
2.03
1.71
1.70
1.22
1.21
1.05
1.05
1.03
0.82
0.81
1.8
2.0
2.3
1.8
1.4
1.0
1.3
2.0
1.1
2.2
4.3
1.1
3.5
12.3
6.0
12.0 11.5 11.0 10.5 10.0 9.5 9.0 8.5 8.0 7.5 7.0 6.5 6.0 5.5 5.0 4.5 4.0 3.5 3.0 2.5 2.0 1.5 1.0 0.5 0.0 -0.5

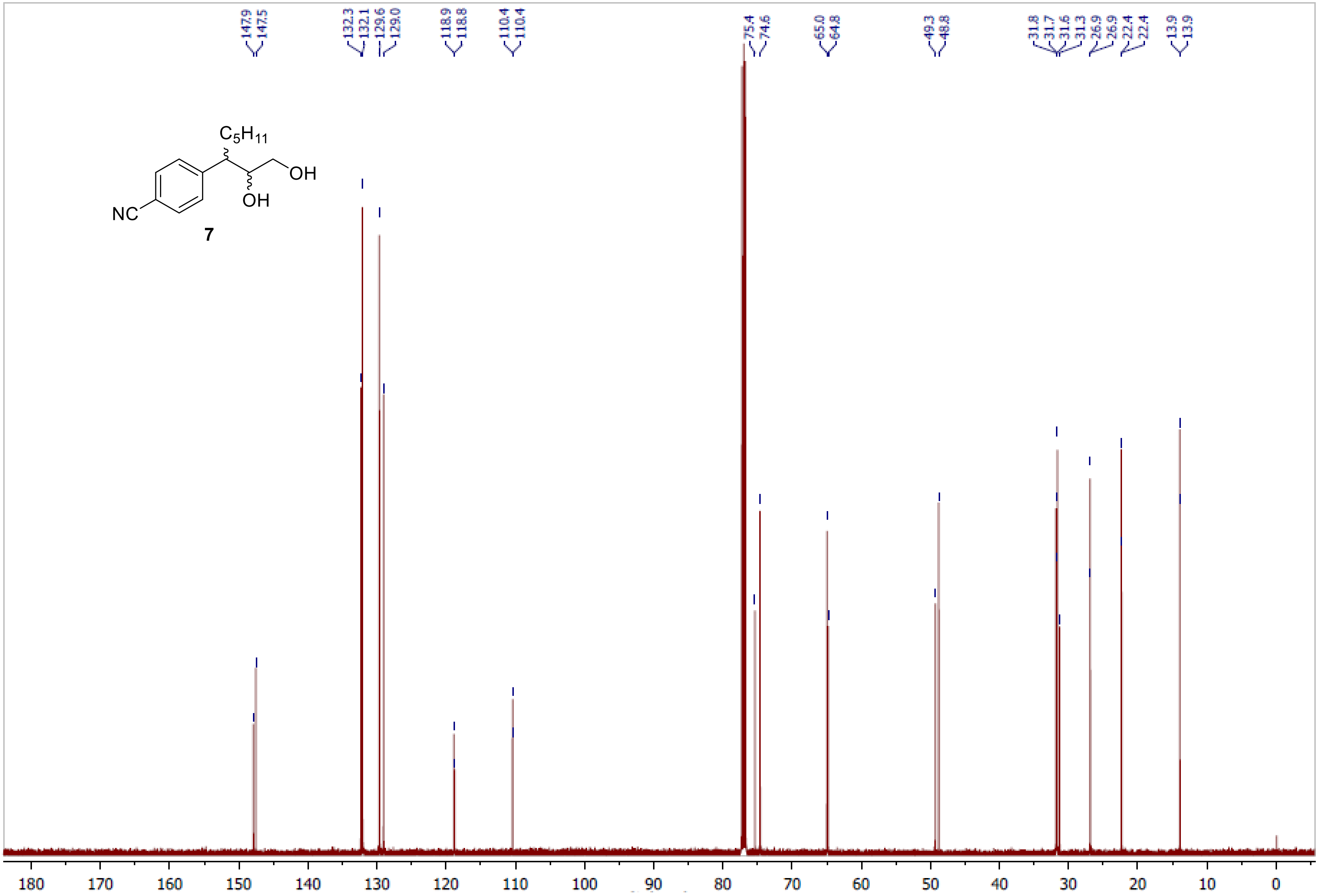
147.9
147.5
132.3
132.1
129.6
129.0
118.9
118.8
110.4
110.4
75.4
74.6
65.0
64.8
49.3
48.8
31.8
31.7
31.6
31.3
26.9
26.9
22.4
22.4
13.9
13.9
C5H11
OH
OH
NC
7
180
170
160
150
140
130
120
110
100
90
80
70
60
50
40
30
20
10
0

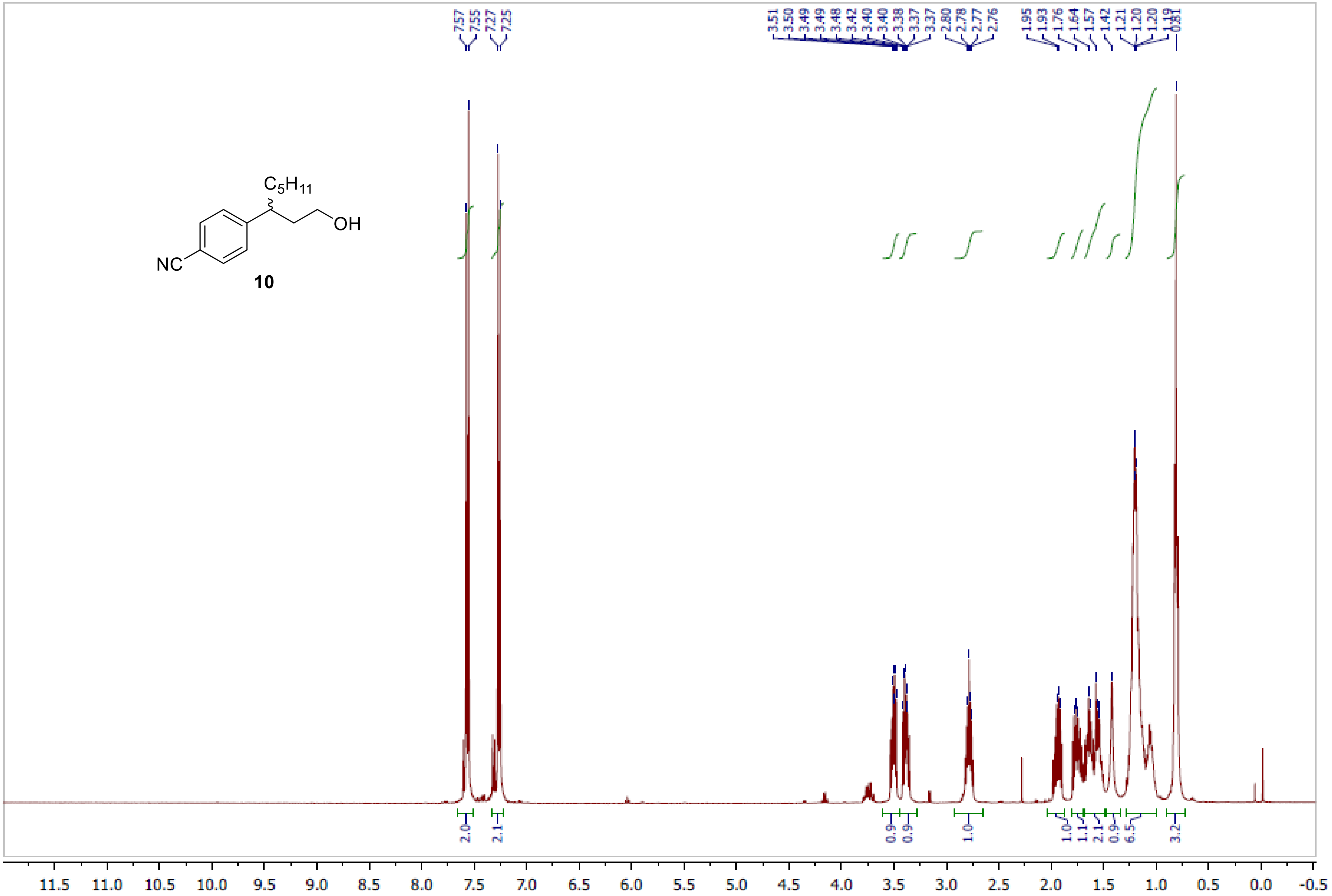
C5H11
OH
NC
10
11.5
11.0
10.5
10.0
9.5
9.0
8.5
8.0
7.5
7.0
6.5
6.0
5.5
5.0
4.5
4.0
3.5
3.0
2.5
2.0
1.5
1.0
0.5
0.0
-0.5

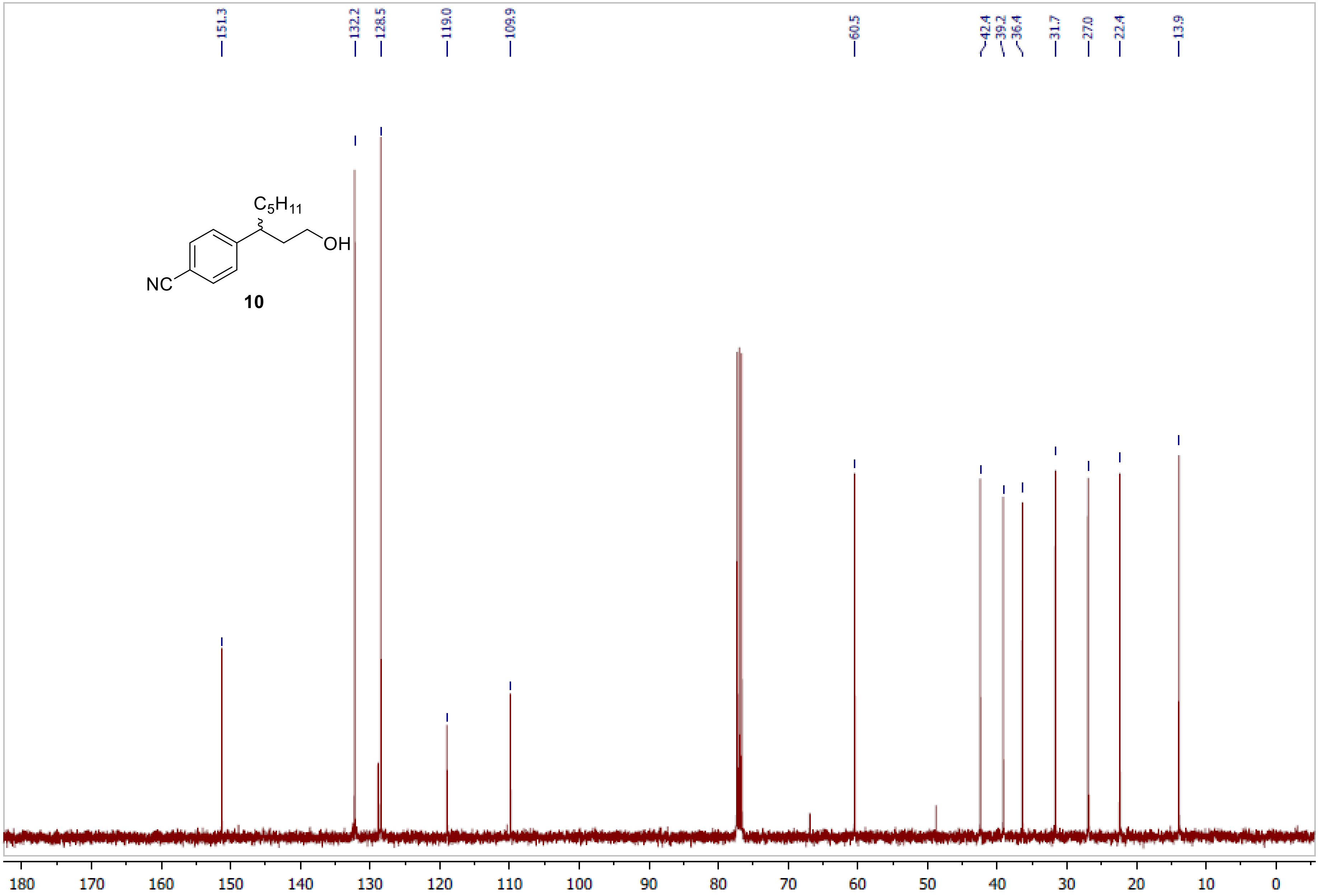
151.3
132.2
128.5
119.0
109.9
60.5
42.4
39.2
36.4
31.7
27.0
22.4
13.9
C5H11
OH
NC
10
180 170 160 150 140 130 120 110 100 90 80 70 60 50 40 30 20 10 0

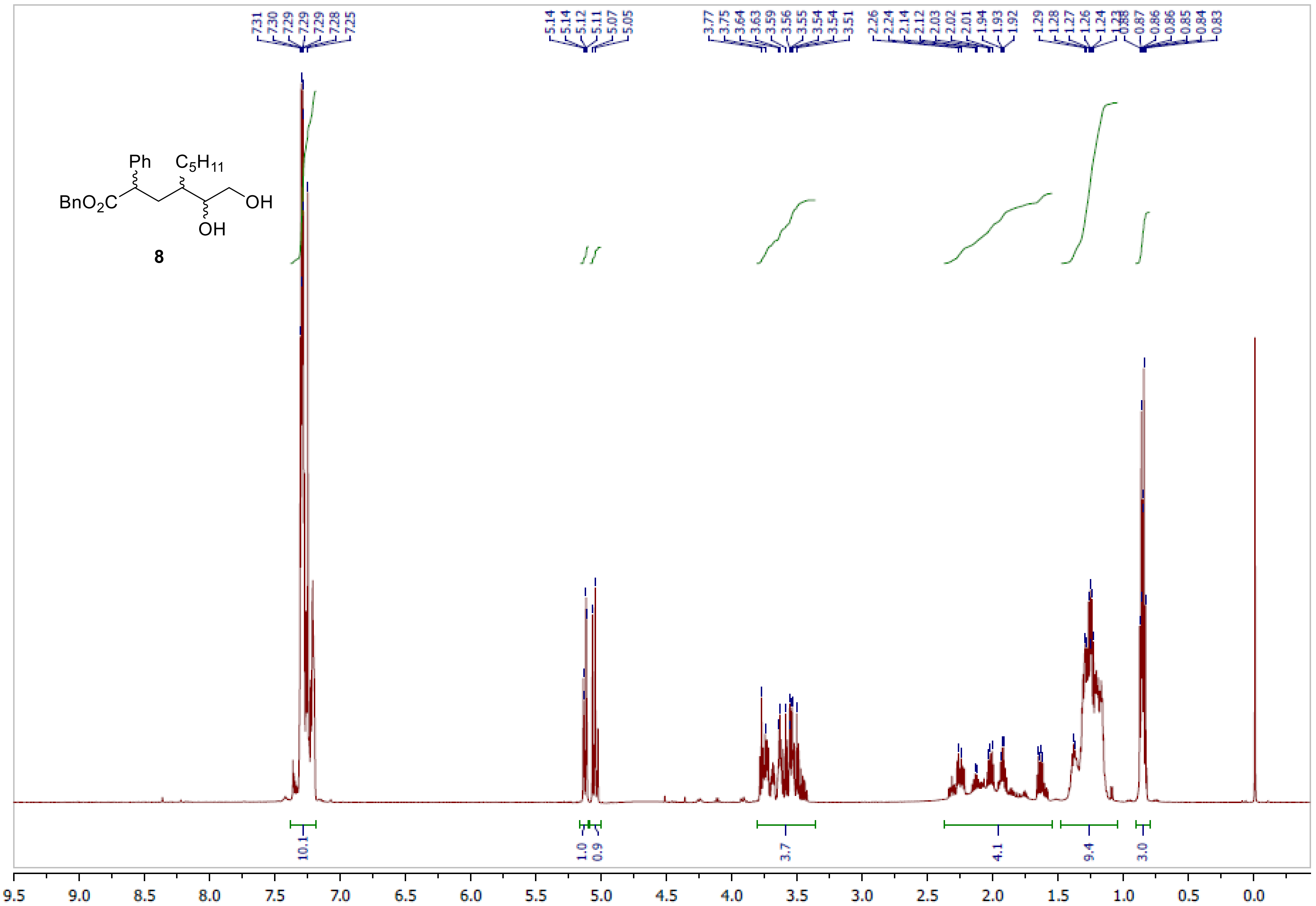
Ph
C5H11
BnO2C
OH
OH
8
7.31 7.30 7.29 7.29 7.29 7.29 7.28 7.25
5.14 5.14 5.12 5.11 5.07 5.05
3.77 3.75 3.64 3.63 3.59 3.56 3.55 3.54 3.54 3.51
2.26 2.24 2.14 2.12 2.03 2.02 2.01 1.94 1.93 1.92
1.29 1.28 1.27 1.26 1.24 1.23
0.88 0.87 0.86 0.86 0.85 0.84 0.83
10.1
1.0
0.9
3.7
4.1
9.4
3.0
9.5 9.0 8.5 8.0 7.5 7.0 6.5 6.0 5.5 5.0 4.5 4.0 3.5 3.0 2.5 2.0 1.5 1.0 0.5 0.0

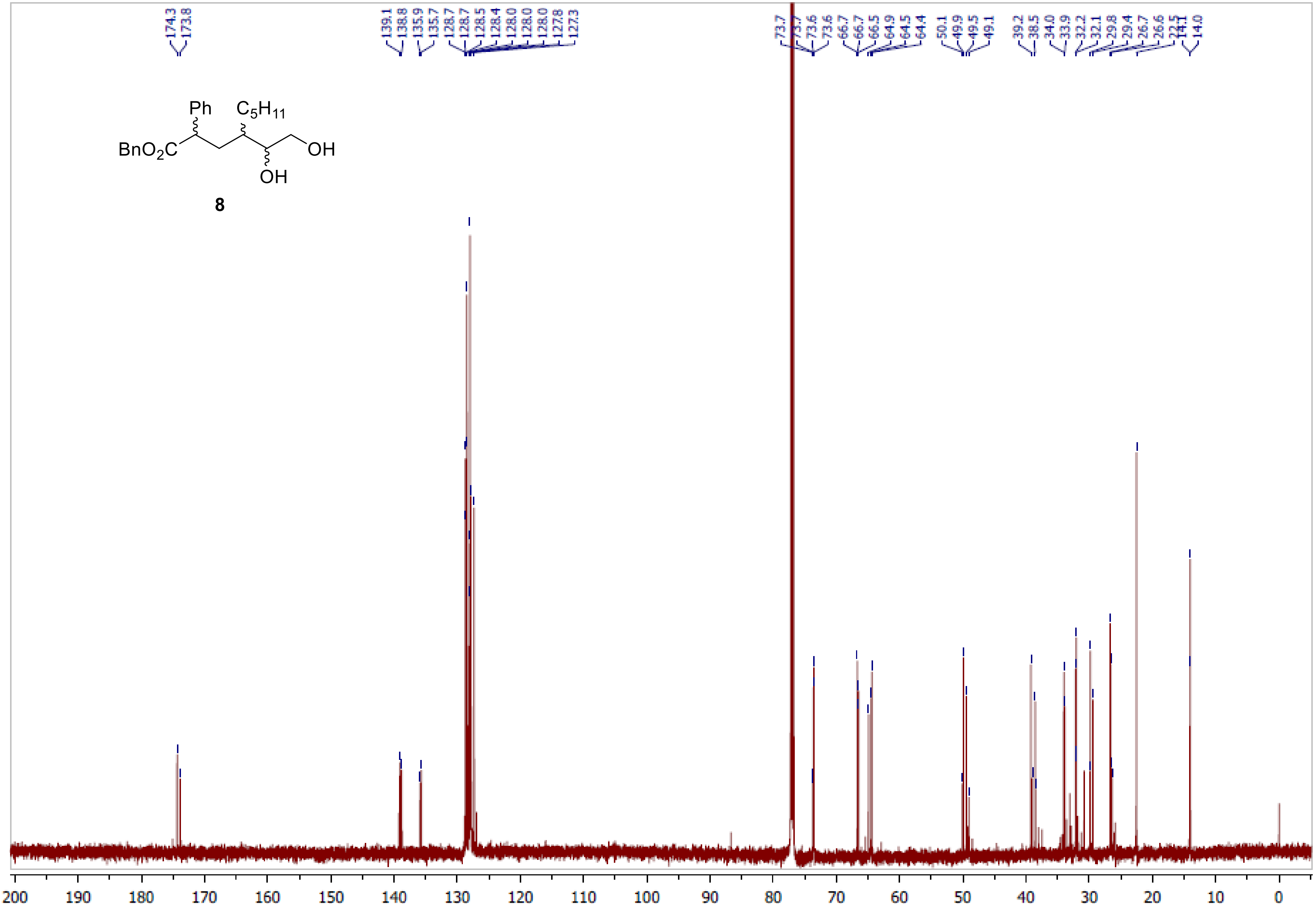
174.3
173.8
139.1
138.8
135.9
135.7
128.7
128.7
128.5
128.4
128.0
128.0
128.0
127.8
127.3
73.7
73.7
73.6
73.6
66.7
66.7
66.5
64.9
64.5
64.4
50.1
49.9
49.5
49.1
39.2
38.5
34.0
33.9
32.2
32.1
29.8
29.4
26.7
26.6
22.5
14.1
14.0
Ph
C_5H_{11}
BnO_2C
OH
OH
8
200 190 180 170 160 150 140 130 120 110 100 90 80 70 60 50 40 30 20 10 0

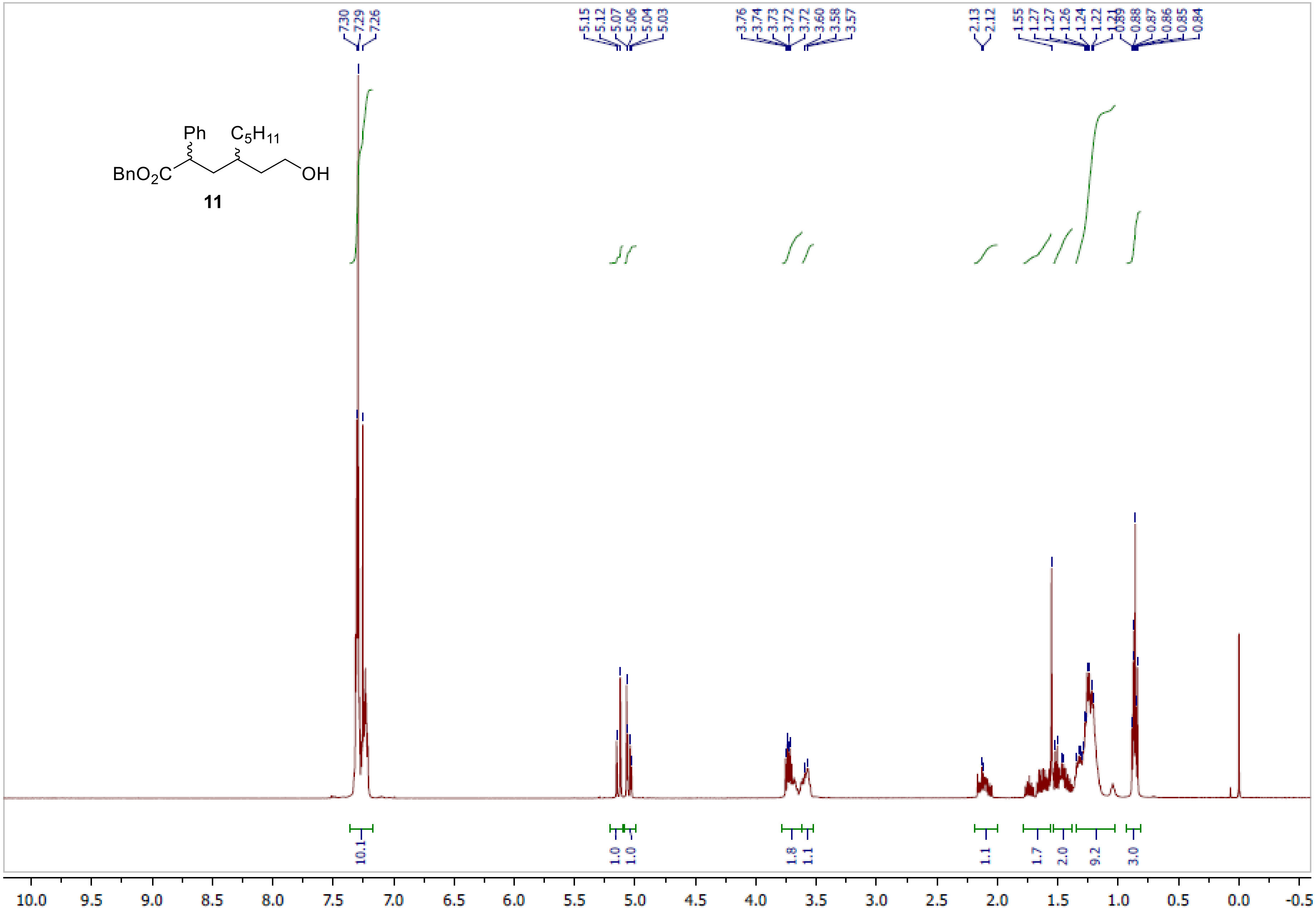
Ph
C5H11
BnO2C
OH
11
7.30
7.29
7.26
5.15
5.12
5.07
5.06
5.04
5.03
3.76
3.74
3.73
3.72
3.72
3.60
3.58
3.57
2.13
2.12
1.55
1.27
1.27
1.26
1.24
1.22
1.21
0.89
0.88
0.87
0.86
0.85
0.84
10.1
1.0
1.0
1.8
1.1
1.1
1.7
2.0
9.2
3.0
10.0
9.5
9.0
8.5
8.0
7.5
7.0
6.5
6.0
5.5
5.0
4.5
4.0
3.5
3.0
2.5
2.0
1.5
1.0
0.5
0.0
-0.5

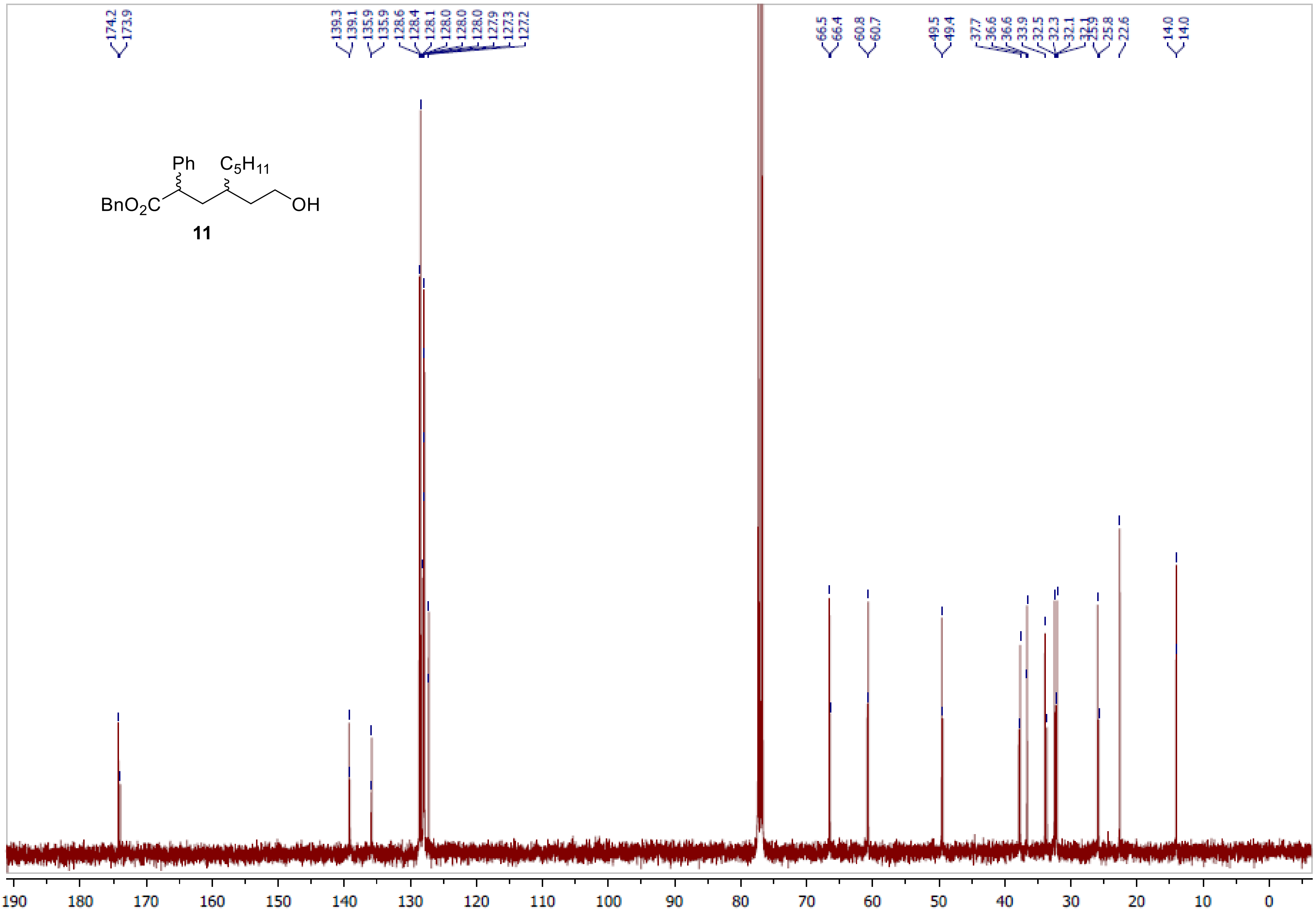
174.2
173.9
139.3
139.1
135.9
135.9
128.6
128.4
128.1
128.0
128.0
128.0
127.9
127.3
127.2
66.5
66.4
60.8
60.7
49.5
49.4
37.7
36.6
36.6
33.9
32.5
32.3
32.1
32.1
25.9
25.8
22.6
14.0
14.0
Ph
C_5H_{11}
BnO_2C
OH
11
190 180 170 160 150 140 130 120 110 100 90 80 70 60 50 40 30 20 10 0

6. References

S1. Silvi, M.; Verrier, C.; Rey, Y. P.; Buzzetti, L.; Melchiorre, P. Nat. Chem. 2017, 9, 868–873. doi:10.1038/nchem.2748

S2. Pirnot, M. T.; Rankic, D. A.; Martin, D. B. C.; MacMillan, D. W. C. Science 2013, 339, 1593–1596. doi:10.1126/science.1232993

S3. Terrett, J. A.; Clift, M. D.; MacMillan, D. W. C. J. Am. Chem. Soc. 2014, 136, 6858–6861. doi:10.1021/ja502639e
